# Mott Transition and Magnetism in Rare Earth Nickelates and its Fingerprint on the X-ray Scattering

**DOI:** 10.1038/s41598-017-10374-2

**Published:** 2017-09-04

**Authors:** Kristjan Haule, Gheorghe L. Pascut

**Affiliations:** Department of Physics and Astronomy, Rutgers University, Piscataway, NJ 08854 United States

## Abstract

The metal-insulator transition (MIT) remains among the most thoroughly studied phenomena in solid state physics, but the complexity of the phenomena, which usually involves cooperation of many degrees of freedom including orbitals, fluctuating local moments, magnetism, and the crystal structure, have resisted predictive *ab-initio* treatment. Here we develop *ab-initio* theoretical method for correlated electron materials, based on Dynamical Mean Field Theory, which can predict the change of the crystal structure across the MIT at finite temperature. This allows us to study the coupling between electronic, magnetic and orbital degrees of freedom with the crystal structure across the MIT in rare-earth nickelates. We predict the electronic free energy profile of the competing states, and the theoretical magnetic ground state configuration, which is in agreement with neutron scattering data, but is different from the magnetic models proposed before. The resonant elastic X-ray response at the K-edge, which was argued to be a probe of the charge order, is theoretically modelled within the Dynamical Mean Field Theory, including the core-hole interaction. We show that the line-shape of the measured resonant elastic X-ray response can be explained with the “site-selective” Mott scenario without real charge order on Ni sites.

## Introduction

Metal-insulator transition (MIT) in transition metal oxides is usually associated with a large Hubbard Coulomb interaction *U* on transition metal ion, which strongly impedes electron motion, as it costs an energy *U* to add an extra electron to any given site. Consequently electrons become localized on the transition metal ion, and hence form a fluctuating moment, which possesses a large entropy that is being released at low temperature by emergence of a long range magnetic order. But most MITs are much more complex than that, and require cooperation of several degrees of freedom, including the subtle change of the crystal structure to tune the hybridization with the oxygen, the modulation of the strength of the fluctuating moments and orbital occupations. In *ab-initio* modeling, this requires one to optimize the crystal structure to the correlated electronic state as an external parameter is varied.

**Figure 1:**
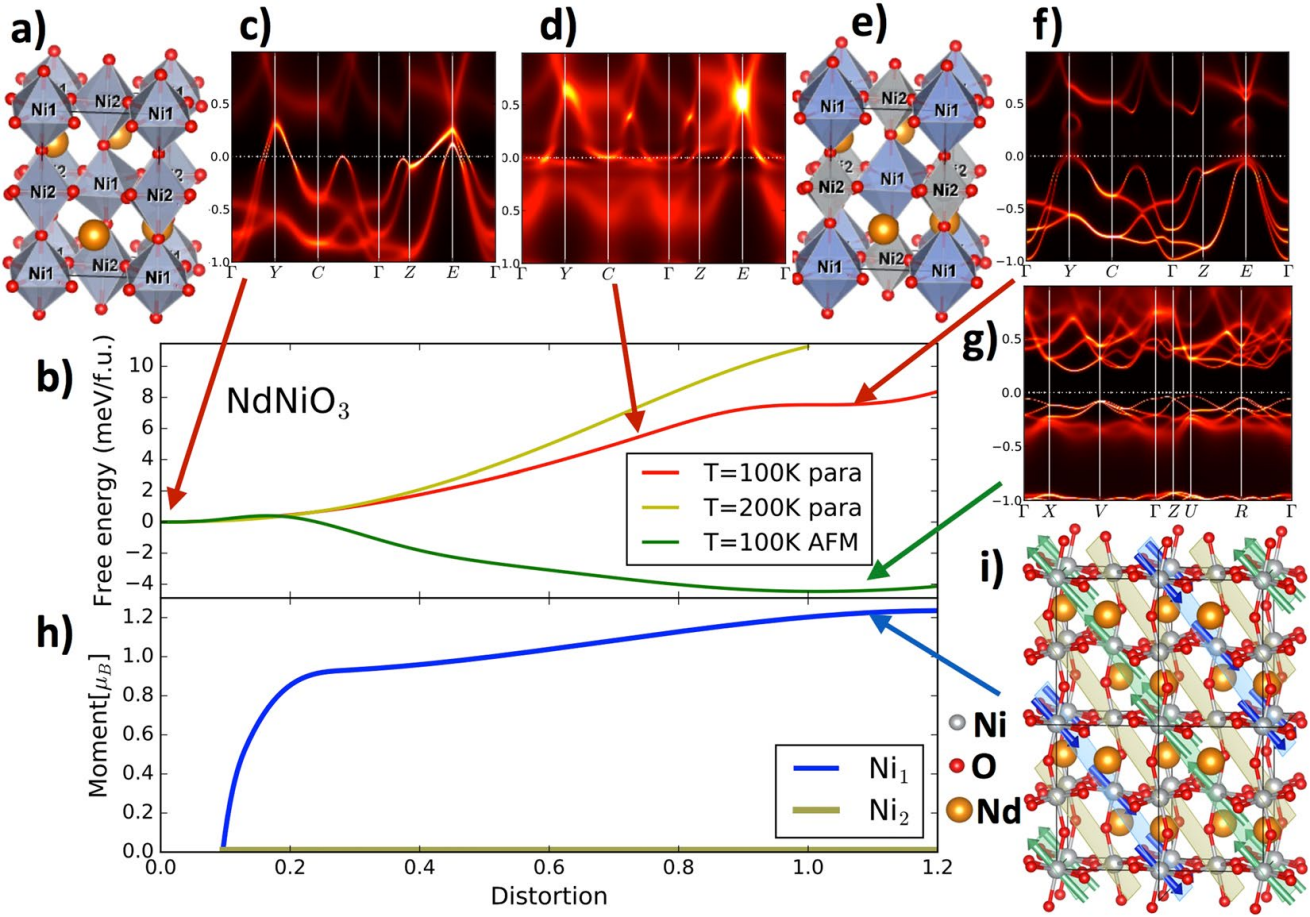
Energetics and magnetism of NdNiO_3_. (**a**) The crystal structure of the metallic NdNiO_3_ stable above $T\gtrsim 200\,\,{\rm{K}}$. (**b**) The electronic free energy of theoretical paramagnetic solution, and antiferromagnetic (AFM) solution as a function of distortion (i.e., linear interpolating between two local minima). (**c**) Spectral function of the paramagnetic metallic state stable at high *T*, (**d**) spectral function of a metastable state at 80% of distortion, (**e**) distorted structure of the insulating state, (**f**) spectral function of the paramagnetic insulating solution at the low *T* equilibrium structure, (**g**) spectral function of the AFM solution (**h**) magnetic moment of the two nickel ions in the AFM state, (**i**) theoretically determined magnetic configuration of the ground state. The planes in (1, 0, 1) direction contain three types of Ni ions: the green (blue) planes contain Ni_1_ atoms with magnetic moments pointing up (down), while the yellow planes contain Ni_2_ atoms which carry no magnetic moment.

The MIT in *R*NiO_3_^1^ is accompanied by the structural transition in which the high-temperature metallic phase, with the orthorhombic (Pbnm) structure (see Fig. 1a), is transformed to the low-temperature insulating phase of monoclinic (P2_1_/n) structure. In the latter, the alternating NiO_6_ octahedra are expanded and compressed in a rocksalt-pattern distortion (see Fig. 1e)^2, 3^. The transition is accompanied by the antiferromagnetic ordering, which occurs simultaneously with the MIT in Nd and Pr compound (*R* = Nd, Pr) and at lower temperature for the smaller rare-earth ions (*R* = Sm and beyond).

The structurally distorted monoclinic ground state is very susceptible to small changes of external parameters and can be tuned by pressure^1^, strain^4–7^, reduced dimensionality^8, 9^ or by layering it in heterostructures^10–12^, hence it has attracted a lot of attention recently.

The leading interpretation for the origin of the MIT is a charge disproportionation (CD) on the Ni sites, in which Ni^3+^ ions disproportionate into sites with excessive and deficient charge (3*d* ^7^3*d* ^7^ → 3*d* ^7+*δ*^3*d* ^7−*δ*^). Such charge order would result in different energy positions of core levels on the two inequivalent Ni ions due to electrostatic effect, which can be probed by the hard resonant elastic X-ray scattering (RXS) through measuring the 1*s* to 4*p* transitions. In ref. 13 it was estimated that the charge order is approximately 2*δ* ≈ 0.42*e* ^13^, based on the 1*s* − 4*p* energy difference of around 0.9 *eV* for the two inequivalent Ni ions. Similar conclusion was reached by numerous other resonant scattering techniques^3, 14–17^. This view has been challenged theoretically, since the *ab-initio* calculations predict very small rearrangement of electronic charge across the transition^18, 19^. On the other hand, the weak coupling theories are supportive of this picture, but also emphasize the cooperation of charge and spin-density wave, with the latter being the driving force of the MIT in NdNiO_3_ and PrNiO_3_^20, 21^.

The alternative explanation posits that Ni experience a “negative charge transfer energy” and consequently is found in a very different *d* ^8^ valence state with compensating holes on the oxygen sites^22, 23^. The compressed octahedra contains Ni *d* ^8^ ion and two ligand holes and the three bind into net zero spin producing unusual continuum of particle-hole excitations^23^, while in the expanded octahedra Ni *d* ^8^ ion is in high-spin *S* = 1 state^24^. Theoretical studies which assume such negative charge transfer energy found a novel state dubbed “site-selective” Mott phase^25, 26^. In this picture the Ni ions in the expanded octahedra undergo the usual Mott transition with two holes on Ni giving rise to a very strong *S* = 1 local moment, while the electrons in the compressed octahedra bind with the (primarily oxygen) states near the Fermi level, and the resulting bonding-antibonding gap opens up, similarly to the band gap of a Kondo insulator^25, 27^.

Although this very appealing picture is accumulating strong support, many fundamental questions remain: (i) How to reconcile the RXS experiments, which require CD, with the picture of “site-selective” Mott transition. (ii) In the seminal work on “site-selective” Mott transition^25^, the physical *d* ^8^ valence was reached by adjusting the onsite energy through an ad-hoc double-counting adjustment, in which Coulomb *U* in the interaction and double-counting were different, in order to reach the “negative charge transfer energy” regime. Similarly, in cluster calculations^24^ the model parameters are chosen such that Ni is found in 3*d* ^8^ configuration. Since the exact double-counting between the Dynamical Mean Field Theory and Density Functional Theory has been derived recently^28^, the assumption of nickel 3*d* ^8^ valence can now be checked without resorting to any *a-priori* assumption on Ni valence. (iii) The propagating vector of the antiferromagnetic order has been unambiguously determined by the neutron scattering^29^, while the precise magnetic configurations was challenging to constrain, and different experiments were interpreted in terms of conflicting models of collinear^30, 31^ and non-collinear^32^ magnetic order. On the other hand, the *ab-initio* electronic structure methods are not supportive of so far proposed models, and suggest that ferromagnetic state is favored compared to proposed antiferromagnetic orders^25, 26^. iv) Many experiments on the Pr and Nd compound^33^ were interpreted in terms of an itinerant picture^20, 21^ in which the spin-density wave drives the MIT. An important question arises: is the magnetic long range order necessary for the MIT in these systems, or, is the Neel order just a consequence of the MIT and it is just a way in which the existing local moments release their entropy.

To address these issues, we use *ab-initio* theoretical method for correlated electron materials, based on combination of dynamical mean field theory (DMFT) and density functional theory (DFT)^34^, in its real space embedded form^35^, which avoids downfolding. To address the issue of Ni valence, we use recently derived exact double-counting between the DFT and DMFT methods^28^. To successfully address the energetics of different competing states and to determine the ground state of the system, it is crucial to theoretically determine the optimized crystal structure, and for this we use recent implementation of forces within DFT-DMFT^36^.

We checked that within this theoretical approach LaNiO_3_ remains paramagnetic metal at least down to 50 *K* and does not show any sign of long range order, in agreement with experiment. On the other hand NdNiO_3_ shows the existence of three phases, the paramagnetic insulating, the antiferromagnetic insulating and the paramagnetic metallic phase. In Fig. 1 we show the energetics of these phases as predicted by the theory. The paramagnetic metallic phase is stable above 200 *K*. Its spectra is plotted in Fig. 1c. The crystal structure in this phase is fully relaxed within the DFT-DMFT theory, and its predicted structural parameters are in excellent agreement with the experiment (see Table 1). For comparison we show the GGA relaxation of the structure, which shows three times larger disagreement with experiment. When the temperature is lowered to above 100 K a first sign of structural instability occurs, as shown in Fig. 1b. The electronic free energy curve of the paramagnetic phase develops a local minimum in the P2_1_/n structure, where oxygen octahedra around Ni_1_ sites are expanded, and octahedra around Ni_2_ sites are compressed. Using the technology to calculate forces^36^, we optimized the structural parameters in this phase (see Supplementary chapter I). In the local minimum, the Mott gap opens up on Ni_1_ atom, while Ni_2_, through strong hybridization with the environment, splits bands such that the band gap opens at the Fermi level, all consistent with the “site-selective” Mott transition scenario^25^ (see Fig. 1f). Just slightly away from this local minimum (80–90% distortion), the insulator breaks down and strongly incoherent metallic state appears (Fig. 1d).

**Table 1:** Optimized atomic positions in the metallic and insulating state of NdNiO_3_.

Pbnm	Exp.	DMFT-PARA	GGA
Ni	(0.000, 0.000, 0.500)	(0.000, 0.000, 0.500)	(0.000, 0.000, 0.500)
O_1_	(0.216, 0.287, 0.539)	(0.214, 0.287, 0.539)	(0.207, 0.294, 0.547)
O_2_	(0.569, 0.490, 0.750)	(0.573, 0.490, 0.750)	(0.591, 0.477, 0.750)
Nd	(0.496, 0.035, 0.750)	(0.491, 0.044, 0.750)	(0.488, 0.058, 0.750)
$\sqrt{\langle {(r-{{\rm{r}}}_{\exp })}^{2}\rangle }$		0.0056	0.0190
**P2 1/n**	**Exp**	**DMFT-AFM**	**GGA + U**
Ni_1_	(0.000, 0.000, 0.000)	(0.000, 0.000, 0.000)	(0.000, 0.000, 0.000)
Ni_2_	(0.000, 0.000, 0.500)	(0.000, 0.000, 0.500)	(0.000, 0.000, 0.500)
O_1_	(0.575, 0.487, 0.752)	(0.574, 0.489, 0.750)	(0.595, 0.475, 0.755)
O_2_	(0.214, 0.276, 0.527)	(0.209, 0.285, 0.540)	(0.198, 0.291, 0.549)
O_3_	(0.719, 0.204, 0.447)	(0.717, 0.210, 0.460)	(0.711, 0.198, 0.452)
Nd	(0.493, 0.039, 0.750)	(0.493, 0.044, 0.750)	(0.489, 0.056, 0.750)
$\sqrt{\langle {(r-{{\rm{r}}}_{\exp })}^{2}\rangle }$		0.0091	0.0180

Experimental structure is from ref. 2. The GGA and GGA + U structure is from ref. 33.

In the Pbnm structure (zero distortion in Fig. 1b,h) the fluctuating moments are present, but they are not strong enough to allow for the long range magnetic order, hence the system resolves its excess entropy in the Fermi liquid state at low temperature. Once the Ni_1_ hybridization is reduced a bit due to small increase of the oxygen octahedra (around 10% distortion), the correlations on Ni_1_ become strong enough so that the static magnetic moment appears (see Fig. 1h). These correlations are primarily driven by the strong Hund’s coupling on Ni ion, which aligns two holes on the Ni_1_ site, but the static ordered moment is only about 2/3 of the maximum moment for spin *S* = 1 state. The resulting magnetic configuration, predicted by the present theory, is displayed in Fig. 1i. The magnetic unit cell quadruples, and the magnetic moment of Ni_1_ ions in the parallel planes in (1, 0, 1) direction are ferromagnetically aligned. The static moments on Ni_2_ however remains exactly zero, as the fluctuating moment on Ni_2_ gets even reduced in the distorted (P2_1_/n) structure, and the strong bonding with the surrounding oxygen concomitant with the appearance of the band gap, prevents any static moment on that site. Every second Ni plane thus carries magnetic moment, and those Ni_1_ planes couple antiferromagnetically. This ordering of moments on Ni_1_ sublattice coincides with the proposed model deduced from the neutron scattering^30^ and resonant soft X-ray diffraction^32^, but it differs from both models due to Ni_2_ sites. In the proposed neutron-scattering model^30^ Ni_2_ moments were arranged antiferromagnetically within a single (1, 0, 1) plane, while in soft X-ray diffraction model^30^, Ni_2_ moments were arranged ferromagnetically, but 90 degrees rotated with respect to Ni_1_ moments, so that the resulting magnetic structure is non-collinear. The magnetic long-range solutions in the DFT-DMFT theory can not sustain finite static moment on Ni_2_, and we show in chapter V of the Supplementary that the theoretical magnetic configuration fits the neutron scattering data as good as the proposed model of ref. 30. Our proposed magnetic configuration is also consistent with the inelastic neutron scattering result, which showed that all Nd ions experience similar Weiss field^37^.

Finally, the gain in free energy is considerable once the magnetic long range order is turned on, hence this magnetic order displayed in Fig. 1i is the theoretical ground state of the displayed unit cell. Table 1 lists the optimized structure in the magnetic state, which shows almost no difference as compared to the paramagnetic structure in P2_1_/n symmetry (given in Supplementary chapter III). From Fig. 1b we can also conclude that the magnetism is not necessary for the metal-insulator transition, but in NdNiO_3_, this paramagnetic insulator appears metastable, and energy gain due to long range magnetic order helps to stabilizes the insulating state. In Supplementary material (chapter II) we show that for smaller rare earth ion (LuNiO_3_) the paramagnetic insulating state is stable at 100 *K* in the absence of magnetism. Magnetism is thus just an efficient way to release the large entropy of fluctuating moment on Ni_1_ sites, which are formed with a help of much stronger Hund’s coupling mechanism.

**Figure 2:**
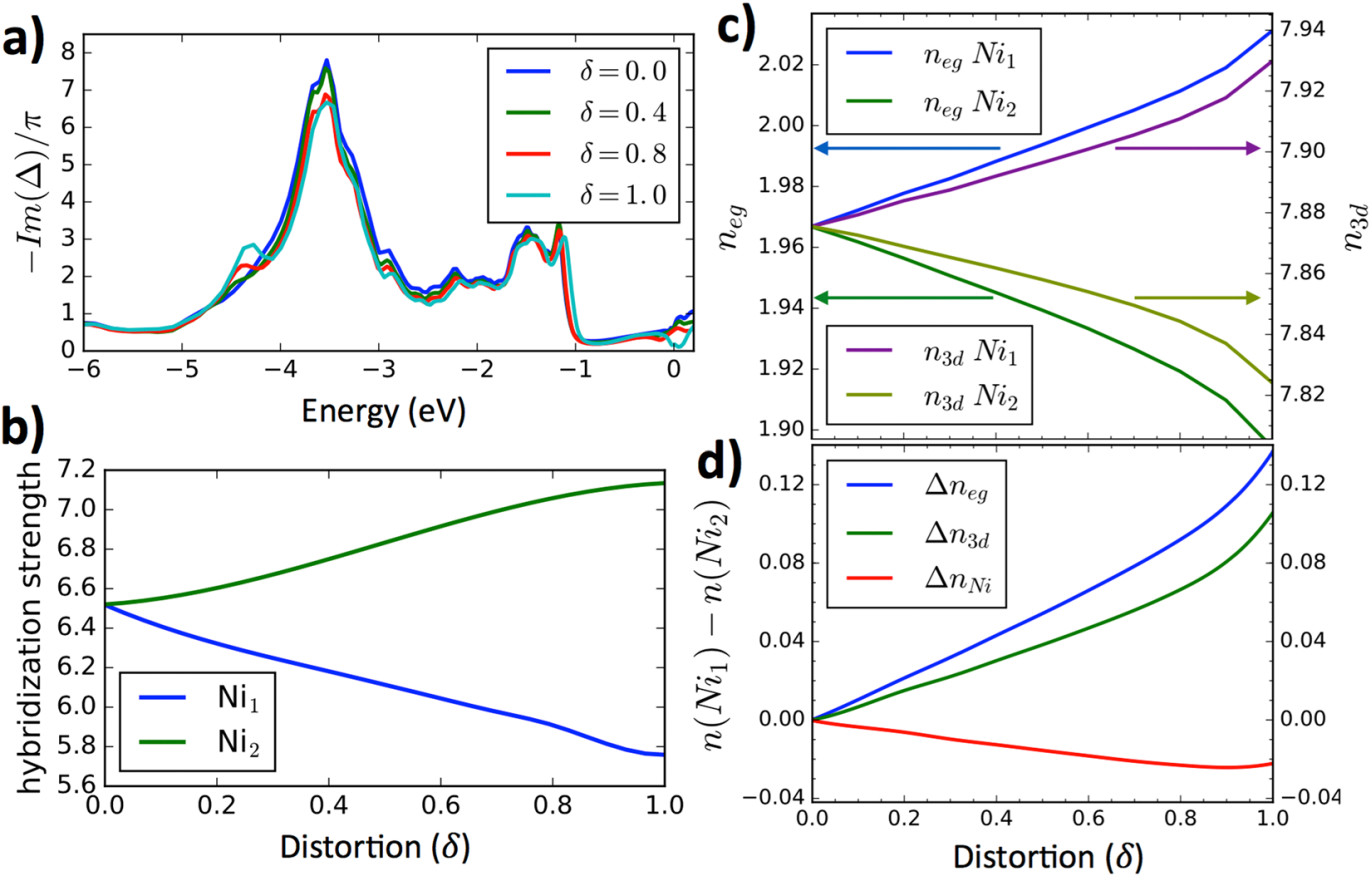
Hybridization and charge of Ni ions. (**a**) Energy dependent hybridization function of the Ni_1_ ion at few values of the distortion parameter *δ* ∈ (0, 1). (**b**) The integral of the hybridization function (in the displayed energy window) as a function of distortion parameter *δ*. (**c**) The charge density on the two Ni atoms corresponding to the *e*_*g*_ orbitals and the entire 3*d* shell. (**d**) The difference of the charge between Ni_1_ and Ni_2_ in the *e*_*g*_ orbital, in the 3*d* shell, and in the entire muffin-tin sphere corresponding to Ni atoms.

While the large Hund’s coupling is essential for the appearance of strong local moments on Ni_1_ sites, the MIT in these materials is tuned by the reduced hybridization on Ni_1_ sites, displayed in Fig. 2a and b. It decreases for about 10% in the bond-disproportionate structure, and this is sufficient for a Mott localization of electrons on Ni_1_ site. Notice that the largest contribution to the hybridization comes from nickel-oxygen overlap, and its reduction is mostly concentrated at the energy of the center of the oxygen states (see Fig. 2a around −3.5 eV). On Ni_2_ sites however, the hybridization increases almost as much as it decreases on Ni_1_ sites (see Fig. 2b), but because the hybridized Ni_2_ and oxygen states in this crystal structure form a band insulator, this increased overlap does not collapse the insulating gap. On the basis of this calculation, we predict that the material would become a canonical Mott insulator if hybridization on both sites gets as small as on Ni_1_ in P2_1_/n structure, which might be possible to achieve in some thin heterostructures of this material^12^.

In Fig. 2c we display the electron charge on Ni ions versus distortion, as obtained by projecting the electron charge to a muffin-tin sphere around each Ni atom of size ≈2 a.u. We notice that there are approximately 2 eg electrons on each site, and approximately 8 electrons in the 3*d* shell, which corresponds to Ni 3*d* ^8^ configuration, as previously postulated in refs 25, 27 and 38, and hence our exact double-counting thus proves the correctness of the negative charge transfer picture for these nickel compounds. We also notice that Ni_1_ (Ni_2_) sites with large (small) octahedra gain (loose) some *eg* electronic charge with structural distortion, and the difference of the *eg* charge on the two Ni sites becomes of the order of 0.137 electrons in equilibrium P2_1_/n structure. However, the electronic charge in the entire 3*d* shell differs only for ≈0.1 electrons, and when all charge inside muffin-tin sphere on Ni is counted, the charge difference is negligible. Moreover, if we were to construct a very low energy model comprised of only the lowest energy bands, we would completely eliminate all Ni_1_ states, as they are pushed to high energy Hubbard bands, and we would conclude that all low energy holes come from Ni_2_ sites. Hence, we can conclude that the appearance of charge order depends on the type of model considered, and while there is no real charge difference in the spheres around each Ni, the low energy models comprised on Ni eg-states only, should allow for charge order.

The CD model was originally invented to explain the RXS results^13^, which showed a strong energy dependent signal at the weak nuclear Bragg peak (*h*, *k*, *l*), where *h* + *k* + *l* is even, and *l* is odd. If the scattering factor *f* of each Ni atoms is approximated by a spherically symmetric quantity, the resonant part of the structure factor is directly proportional to the difference ${f}_{N{i}_{1}}-{f}_{N{i}_{2}}$ (see Supplementary). A strong X-ray signal at the Ni resonance can therefore be taken as a direct evidence of a very large difference between the two Ni atoms. In particular, for the X-ray K-edge measurement, this must mean that the energy difference between the core 1*s* state and the valence 4*p* states of the Ni ion is very different on the two inequivalent Ni sites. As the core energy is very sensitive to the amount of the charge on Ni ion, it is generally accepted that the difference in the core energy comes from the different charge accumulated on Ni_1_ and Ni_2_ ions. As our model predicts negligible total charge difference on the two Ni sites, the X-ray scattering needs an alternative explanation.

**Figure 3:**
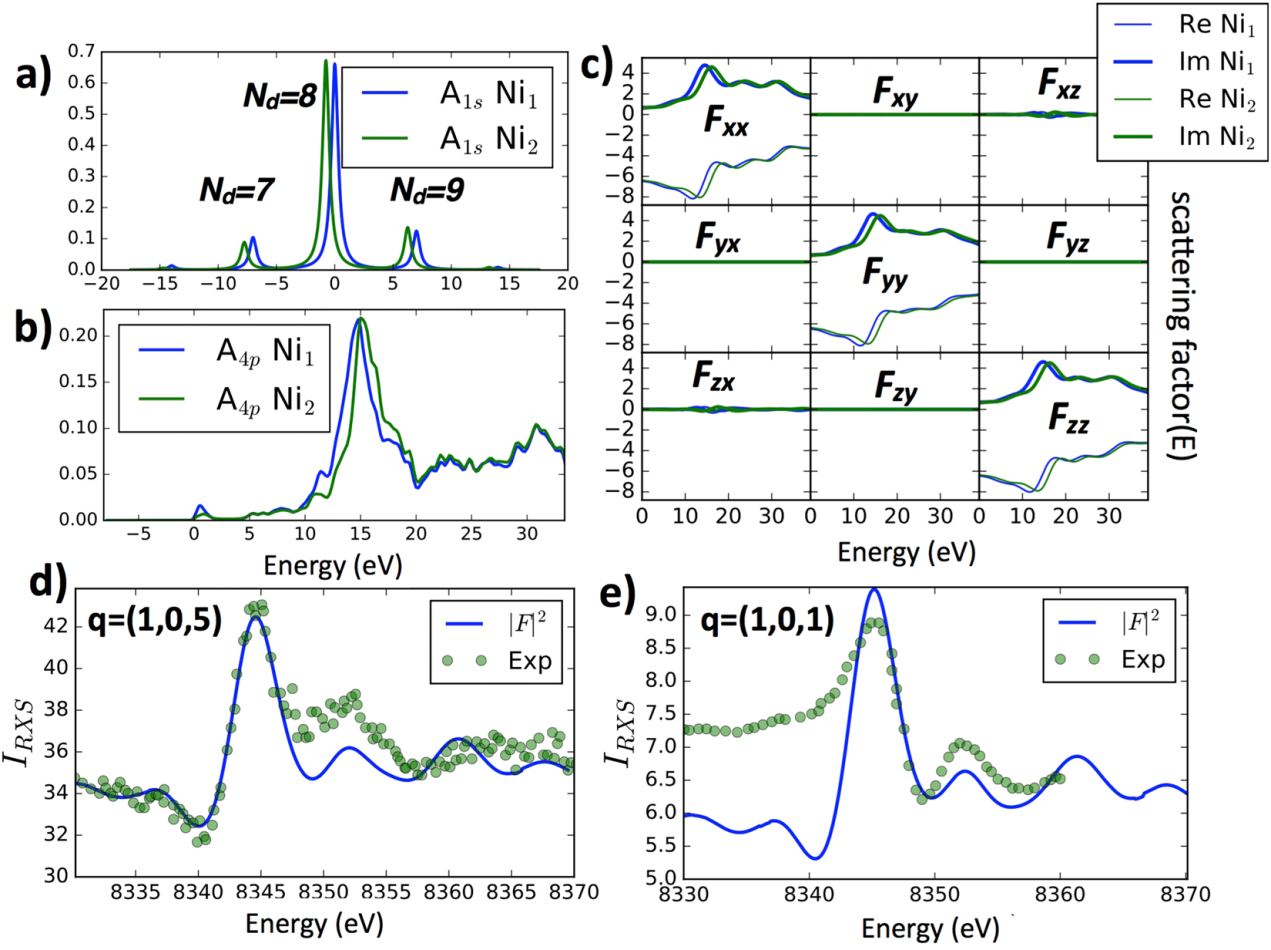
Resonant Elastic X-ray scattering on Ni K-edge. (**a**) The spectral function of the 1*s* core state in the presence of the fluctuating valence of the Ni 3*d* shell. (**b**) Ni 4*p* density of state. (**c**) the energy dependent matrix of the scattering factor, where E means electron units (**d**,**e**) measured and computed X-ray scattering intensity at the two Bragg peaks. Experimental data in (**d**) are reproduced from ref. 13 and in (**e**) from ref. 33.

In Fig. 3a and b we show the calculated spectral function for the Ni 1*s* core and 4*p* valence orbital. The 1*s* spectra on Ni_1_ ion is shifted up compared to Ni_2_ for approximately 0.7 eV, and the 4*p* spectra is shifted in opposite direction for approximately 0.8 eV, resulting in approximately 1.5 eV difference in the 1*s* → 4*p* transition energy on two inequivalent Ni atoms. Such energy difference can explain the occurence of the main peak in the RXS intensity displayed in Fig. 3d,e, hence no charge order is needed for its explanation. However, the multiple peak structure of the intensity can not be explained by only the single-particle effects and the structural distortion. The 4*p* states are very extended and do not appreciably overlap with the core 1*s* states, hence the Coulomb repulsion between the two can be neglected. However, the Ni 1*s* and partially filled 3*d* orbitals have large overlap, therefore the Coulomb repulsion between the two states is comparable to the Coulomb *U* among electrons in the 3*d* shell. In this work, we included such core-hole interaction between the Ni 1*s* and Ni 3*d* states, which takes the form Δ*H* = *U*_*ch*_(*n*_1*s*_ − 2)(*n*_3*d*_ − *n*_3*d*_). We took *U*_*ch*_ = 7 eV, the same as *U* in the 3*d* shell. When such term is included in the Hamiltonian, the core 1*s* orbital experiences different energy when the 3*d* shell is in different valence state. As there are substantial valence fluctuations in this system with finite probability for Ni *d* ^7^ and *d* ^9^ valence, the core state spectra is split into three peaks, roughly separated by *U*_*ch*_. Finally, the scattering factor on each Ni, is computed by convoluting 1*s* and 4*p* spectra (see Supplementary chapter IV) and is displayed in Fig. 3c. The *xy* and *yz* components vanish by the symmetry, and only diagonal and the *xz* components are finite. Moreover, the *xz* component is one order of magnitude smaller than the diagonal components, as consistent with the fact that the *σ* − *π* intensity is two orders of magnitude smaller than *σ* − *σ* scattering intensity^39, 40^ (the off-diagonal component would contribute to the scattering in *σ* − *π* channel). Moreover, the diagonal components have a pre-peak shoulder roughly *U*_*ch*_ below the main peak, and second and third peak roughly *U*_*ch*_ and 2*U*_*ch*_ above the main peak, all consequence of the core-hole interaction. Finally, computing the square of the total structure factor we arrive at the X-ray intensity, displayed in Fig. 3d and e. This is directly compared with the experiment, and we notice that reasonable agreement is achieved without any fitting parameter. We can thus conclude that the inequivalent Ni-sites harboring “site-selective Mott transition” but no real charge order, can explain all important observation in the rare-earth nickelates, including the magnetic long range order consistent with neutron scattering data, and the resonant X-ray intensity in the weak nuclear Bragg peaks, which was previously assumed to be a proof of the electronic charge order.

## Electronic supplementary material

Supplementary Information
