# Supplementary material for: Mott Transition and Magnetism in Rare Earth Nickelates and its Fingerprint on the X-ray Scattering
Source: Sci Rep. 2017 Sep 4;7:10375. doi: 10.1038/s41598-017-10374-2 (PMC5583322; doi:10.1038/s41598-017-10374-2)
Supplement: Supplementary file 1 — Supplementary Information [file 41598_2017_10374_MOESM1_ESM.pdf]

Mott Transition and Magnetism in Rare Earth Nickelates and its Fingerprint on the X-ray Scattering

K. Haule and Gheorghe L. Pascut
Department of Physics and Astronomy, Rutgers University, Piscataway, NJ 08854, United States.
(Dated: May 24, 2017)

Contents

I. The DFT+EDMFT theoretical method 1

II. Metal-insulator transition in $LuNiO_3$ and its comparison to $NdNiO_3$ 2

III. Optimized Crystal Structure of $NdNiO_3$ in para and AFM state 4

IV. X-ray scattering spectroscopy within DFT+EDMFT 4

V. Reanalyzing the neutron powder diffraction experiments and their consistency with proposed magnetic models 9

VI. Technical Details on the neutron powder refinements 13
A. Reproducing published results of Ref. 1 where the crystal structure was believed to be described by Pbnm symmetry 13
B. New results obtained when using the low temperature crystal structure described by $P2_1/n$ symmetry 13

VII. Insights into the neutron magnetic scattering 14
A. Ni moments only 16
1. Ni moments along a high symmetry direction 16
2. Ni moments along $(1, 0, 1)$ and $(-1, 0, 1)$ direction 16
B. Rare earth moments with different propagating vector 17
C. Rare earth moments with $\mathbf{q} = (1, 0, 1)$ propagating vector 17
D. Moments along high symmetry direction 19

VIII. Magnetic X-ray scattering is consistent with DMFT model 19

References 21

I. THE DFT+EDMFT THEORETICAL METHOD

We use the theoretical *ab-initio* method for correlated materials [2], which combines the Density Functional Theory with the Embedded Dynamical Mean Field Theory (DFT+EDMFT) [3, 4] in a functional form [5]. The corresponding functional being extremized is defined in real space [5, 6], and takes the form

$$\Gamma[\{G\}] = \mathrm{Tr}\log(-G) - \mathrm{Tr}((G_0^{-1} - G^{-1})G) + \Phi[\{G\}] + E_{nuclei} \quad (1)$$

where

$$\Phi[\{G\}] = E_H[\rho] + E_{XC}[\rho] + \sum_{\mathbf{R}\in \mathrm{Ni}} \Phi^{DMFT}_{V_{DMFT}}[\{G^{\mathbf{R}}_{local}\}] - \Phi^{DC}_{V_{DMFT}}[\{\rho^{\mathbf{R}}_{local}\}] \quad (2)$$

and E_H, E_{XC} and Φ^{DMFT} are the Hartree, the LDA exchange-correlation functional and the DMFT Luttinger-Ward functional. $\Phi^{DC}_{V_{DMFT}}[\{\rho^{\mathbf{R}}_{local}\}]$ is the exact double-counting [7], computed by solving an auxiliary electron gas problem, interacting by the screened Coulomb repulsion V_{DMFT}. [7] Here the screened interaction V_{DMFT} in the real space is parametrized by $V_{DMFT}(r) = e^{-\lambda r}/(\varepsilon r)$, and λ and ϵ are determined so that they give estimated values for the Slater integrals. We estimate that the Slater monopole term

$F^0 = \langle \phi, \phi | V_{DMFT} | \phi, \phi \rangle$ is 7 eV for Ni atoms, and Hund's coupling $J = 1.1$ eV (corresponding to Slater $F^2 = 9.5$ eV and $F^4 = 5.9$ eV), which uniquely fixes $\lambda \approx 1.52/a.u.$ and $\varepsilon = 1.31$ for our quasi-atomic orbitals $\phi^{\mathbf{R}}(\mathbf{r})$, defined below. We use the Slater form of the Coulomb repulsion in its rotationally invariant form.

In our embedded DMFT approach, the local Green's function is obtained by projection in real space

$$G^{\mathbf{R}}_{local}(\mathbf{r}, \mathbf{r}'; \omega) = \sum_{\alpha,\beta} \langle \mathbf{r} | \phi^{\mathbf{R}}_\alpha \rangle \langle \phi^{\mathbf{R}}_\alpha | G(\omega) | \phi^{\mathbf{R}}_\beta \rangle \langle \phi^{\mathbf{R}}_\beta | \mathbf{r}' \rangle \quad (3)$$

and $\phi^{\mathbf{R}}(\mathbf{r})$ are the $3d$ solutions of the Schroedinger equation on the Ni ions (inside the muffin-tin sphere) using DFT Kohn-Sham potential inside the sphere. As these states are not bound states, the energy at which the Schroedinger equation is solved needs to be chosen properly. We use the Fermi energy as the chosen energy at which the Schroedinger equation is solved to obtain $\phi^{\mathbf{R}}(\mathbf{r})$.

Note that in this approach, the Dyson equation

$$G^{-1}(\mathbf{r}, \mathbf{r}') = G_0^{-1} - V_H - V_{XC} - \Sigma^{DMFT} + \Sigma^{DC}, \quad (4)$$

(obtained by direct extremization of the functional Eq. 1) is solved in the large Hilbert space spanned by the LAPW basis functions, which is very different from many other approaches, which commonly implement DFT+EDMFT in a Wannier basis, and therefore use approximations, such as downfolding or constructing approximate Hubbard Hamiltonians.

The functional derivative of the DMFT functional requires the following self-energy in the Dyson equation

$$\Sigma^{DMFT}(\mathbf{r}, \mathbf{r}') = \sum_{\alpha,\beta} \langle \mathbf{r} | \phi^{\mathbf{R}}_\alpha \rangle \frac{\delta \Phi^{DMFT}[\{G_{local}\}]}{\delta G_{local\,\beta\alpha}} \langle \phi^{\mathbf{R}}_\beta | \mathbf{r}' \rangle (5)$$

hence we calculate the self-energy $\frac{\delta \Phi^{DMFT}[\{G_{local}\}]}{\delta G_{local\,\beta\alpha}} = \Sigma_{\alpha,\beta}$, which is being computed by the continuous time quantum Monte Carlo impurity solver [8]. The (L)APW+lo basis and the Kohn-Sham Hamiltonian $V_H + V_{XC}$ are constructed by the Wien2k algorithm [9]. We use LDA parametrization for the DFT exchange-correlation functional, as it is more precise for heavier atoms.

We also treat Nd atoms with the DMFT, but we use Hubbard-I impurity solver for Nd atoms, as the electrons on these atoms are very localized at 100 K. We found that merely adding Nd atoms to core, as is commonly done, leads to underestimation of the size of Nd ions, and therefore not sufficiently precise predictions of crystal structures. Note also that the correlated Ni-d states are treated dynamically by the DMFT and are simulated at finite temperature, while the itinerant states (from oxygen and Ni-s, etc) are treated within LDA exchange correlation approximation.

The functional form of our DFT+EDMFT method allows us the compute forces on all atoms by taking the analytic derivative of the free energy functional [6]. The implementation of forces [6] is essential for obtaining the equilibrium structures of the method, and computing the free energy difference between competing crystal structures. Namely, taking the structure from experiment, or from DFT optimization, results in theoretical energy of the order of 10-30 meV above the minimum energy, which is much larger than the energy differences we are interested in across metal-insulator transition.

It turns out that the calculation of the force is more stable and less affected by the Monte-Carlo noise than computing the value of the functional itself, as shown in Ref. 6. This is because the free energy requires the calculation of the Φ^{DMFT} functional, which contains the electronic entropy, and can only be computed by an integral over temperature. The force, on the other hand, requires only the self-energy, which can be very efficiently calculated by the Monte Carlo impurity solvers. We thus computed the free energy differences by computing the force along the path between two structures, and we integrated the force, to obtain the free energy along the path between two structures. Using this method, we achieved statistical error bars from Monte Carlo simulation to be smaller than 0.25 meV/f.u.

II. METAL-INSULATOR TRANSITION IN $LuNiO_3$ AND ITS COMPARISON TO $NdNiO_3$

Similarly to $NdNiO_3$ the DFT+EDMFT method predicts a metal insulator transition in other rare-earth nickelates, and in Fig. 1 we are displaying the transition in $LuNiO_3$, which is theoretically particularly simple, as the f-shell is fully filled. In Fig.1 of the main text, and in this Fig. 1 we show the free energy along the path between the two theoretically optimized structures. We are using a linear interpolation between the two structures ($\mathbf{R}^i(d) = \mathbf{R}^i_{Pbnm} + (\mathbf{R}^i_{P2_1/n} - \mathbf{R}^i_{Pbnm})d$, d =distortion).

It is clear from Fig. 1 that below about 500 K the insulating state with bond disproportionation becomes stable, and that a clear double-well free energy profile emerges, typical for a first order phase transitions. For stability of the insulating state, the magnetic long range order is not necessary in $LuNiO_3$, because Lu ion is much smaller than Nd ion, and requires much stronger octahedra rotations, and consequently longer Ni-O bond distance (1.97Å in $LuNiO_3$ versus 1.95Å in $NdNiO_3$). The theoretical disproportionation of the bond lengths of the two octahedra is also larger in LuNiO3, Ni_1 (Ni_2) octahedra have bond-length 2.007 (1.927) as compared to their bond-length in $NdNiO_3$, which is 1.978 (1.917).

At 100 K Pbnm structure is still a local minimum, but extremely shallow, while $P2_1/n$ structure have around 6 meV per formula unit lower energy then Pbnm structure. We also notice that the gap opens in single particle spectra once the system climbs across the barrier and the free energy slopes towards the insulating minimum, hence we clearly see opening of the Mott gap at half distortion

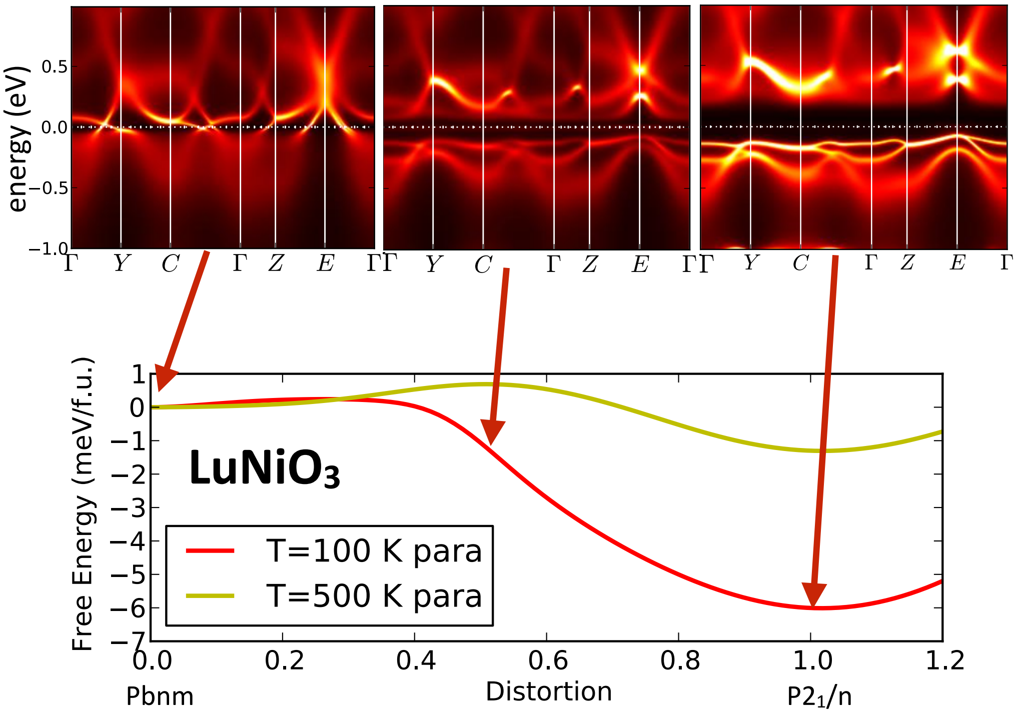

FIG. 1: **Energetics and spectra of $LuNiO_3$** in paramagnetic state at $T = 100\,K$ and $T = 500\,K$. The top row shows the spectral function $A(\mathbf{k}, \omega)$ at 100 K and at three different distortion levels, in the Pbnm structure (zero distortion), half way towards equilibrium structure, and in the equilibrium $P2_1/n$ structure.

towards $P2_1/n$ structure.

We notice in passing that to properly compute the energetics of the metal-insulator transition, it is crucial to optimize the structure in both minima. For example, if one optimizes the $P2_1/n$ (Pbnm) structure within GGA+U (GGA) method, and then uses this structure to compute the DMFT free energy, the resulting free energy is 33 meV/f.u. (25 meV/f.u.) higher than in the optimized structure, which is five-times more that the energy difference between the two structures. For example, in Ref. 10 three different theoretical methods were combines in attempt to compute the total energy profile across the metal insulator transition: to optimize the Pbnm structure GGA was used, to optimize $P2_1/n$ structure GGA+U was used, and to compute the total energy along the path DFT+EDMFT method was used. In this case the path was connecting two structures which are very far from their respective equilibrium, and therefore can not characterize the free energy lanscape of the theoretical solutions.

Finally, let us show the self-energy of the two compounds in the paramagnetic insulating state (see Fig. 2). The larger octahedra containing Ni_1 in the center harbor almost all fluctuating moment, therefore the self-energy has a pole in the Mott gap. On the other hand, Ni_2 ions in the small octahedra are bonded with oxygen, develop a band gap, and show very weak renormalization of the electronic structure, as previously explained in Ref. 11. The fluctuating magnetic moment on Ni_2 sites is negligible. Notice also that orbital splitting is completely negligible in $NdNiO_3$, while it gets somewhat enhanced in $LuNiO_3$.

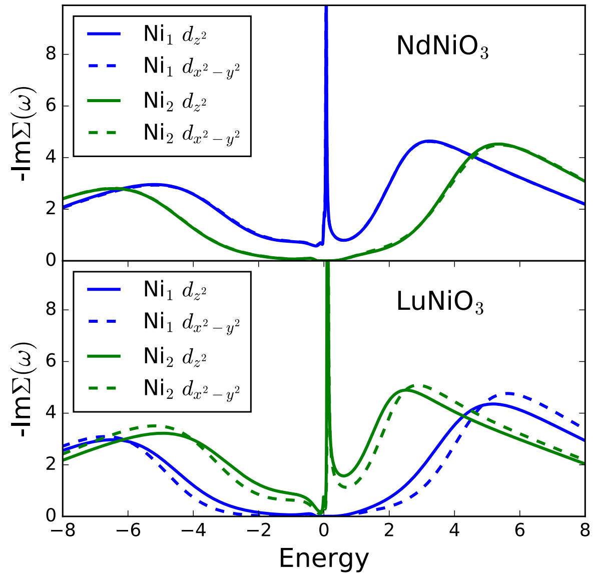

FIG. 2: **The electronic self-energy** in the equilibrium structure at 100 K in the paramagnetic insulating state.

III. OPTIMIZED CRYSTAL STRUCTURE OF $NdNiO_3$ IN PARA AND AFM STATE

In the main text of the paper, we showed the optimized parameters for the $NdNiO_3$ crystal structure in paramagnetic Pbnm symmetry and antiferromagnetic (AFM) $P2_1/n$ symmetry. But the DFT+EDMFT functional has the third solution, the paramagnetic Mott insulating $P2_1/n$ structure, which is a local minimum in Fig.1b of the main text. We had to optimize this structure as well to find the energy barrier between different structures. Here we show all three DMFT optimized structures. The important lesson learned from this optimization is that the AFM and para crystal structures in $P2_1/n$ symmetry are almost identical, hence the magnetic long range order has almost no effect on crystal structure, and the fluctuating moments in Mott state are as good as the order moments in AFM state for optimizing crystal structure. This is consistent with the fact that in late RNiO$_3$ (with R being the late rare earth ion) in which the Mott transition and AFM transition are decoupled, the crystal structure changes abruptly across the Mott transition, but is almost unchanged across the AFM phase transition.

TABLE I: Optimized atomic positions in the metallic and insulating state of $NdNiO_3$. Experimental structure is from Ref. 12. The GGA and GGA+U structure is from Ref. 13.

Pbnm	Exp.	DMFT-PARA		GGA
Ni	(0.000, 0.000, 0.500)	(0.000, 0.000, 0.500)		(0.000, 0.000, 0.500)
O_1	(0.216, 0.287, 0.539)	(0.214, 0.287, 0.539)		(0.207, 0.294, 0.547)
O_2	(0.569, 0.490, 0.750)	(0.573, 0.490, 0.750)		(0.591, 0.477, 0.750)
Nd	(0.496, 0.035, 0.750)	(0.491, 0.044, 0.750)		(0.488, 0.058, 0.750)
$\sqrt{\langle(\mathbf{r}-\mathbf{r}_{exp})^2\rangle}$		0.0056		0.0190
$P2_1/n$	Exp	DMFT-PARA	DMFT-AFM	GGA+U
Ni_1	(0.000, 0.000, 0.000)	(0.000, 0.000, 0.000)	(0.000, 0.000, 0.000)	(0.000, 0.000, 0.000)
Ni_2	(0.000, 0.000, 0.500)	(0.000, 0.000, 0.500)	(0.000, 0.000, 0.500)	(0.000, 0.000, 0.500)
O_1	(0.575, 0.487, 0.752)	(0.575, 0.489, 0.754)	(0.574, 0.489, 0.750)	(0.595, 0.475, 0.755)
O_2	(0.214, 0.276, 0.527)	(0.209, 0.284, 0.540)	(0.209, 0.285, 0.540)	(0.198, 0.291, 0.549)
O_3	(0.719, 0.204, 0.447)	(0.717, 0.209, 0.460)	(0.717, 0.210, 0.460)	(0.711, 0.198, 0.452)
Nd	(0.493, 0.039, 0.750)	(0.491, 0.044, 0.750)	(0.493, 0.044, 0.750)	(0.489, 0.056, 0.750)
$\sqrt{\langle(\mathbf{r}-\mathbf{r}_{exp})^2\rangle}$		0.0090	0.0091	0.0180

IV. X-RAY SCATTERING SPECTROSCOPY WITHIN DFT+EDMFT

The X-ray absorption intensity I_{XAS} and the resonant X-ray scattering intensity I_{RXS} are usually represented in terms of the scattering tensor F in the following way

$$I_{XAS} = -\frac{1}{\pi}\mathrm{Im}[F(\vec{\varepsilon},\vec{\varepsilon'})] \quad (6)$$

$$I_{RXS} = \left|F(\vec{\varepsilon},\vec{\varepsilon'})\right|^2 \quad (7)$$

where the scattering tensor can be broken up into

$$F = F^{Thompson} + F^{res} \quad (8)$$

a sum of non-resonant Thompson part

$$F_{\mathbf{q}}^{Thompson} = r_0\, \vec{\varepsilon'}^*\vec{\varepsilon}\int d^3r\, e^{i\mathbf{qr}}\rho(\mathbf{r}), \quad (9)$$

which is very large, and can not be ignored, and the resonant part

$$F^{res} = \frac{r_0}{4m}\sum_n \frac{\langle g|\vec{\varepsilon'}^*\int d^3r(e^{-i\vec{k'}\vec{r}}\hat{\psi}_c^\dagger(i\hbar\nabla)\hat{\psi}_v + \hat{\psi}_c^\dagger(i\hbar\nabla)\hat{\psi}_v e^{-i\vec{k'}\vec{r}})|n\rangle\langle n|\vec{\varepsilon}\int d^3r(e^{i\vec{k}\vec{r}}\hat{\psi}_v^\dagger(i\hbar\nabla)\hat{\psi}_c + \hat{\psi}_v^\dagger(i\hbar\nabla)\hat{\psi}_c e^{i\vec{k}\vec{r}})|g\rangle}{E_g - E_n + \hbar\omega} \quad (10)$$

where $r_0 = \frac{1}{4\pi\varepsilon_0}\frac{e^2}{mc^2}$, $\vec{\varepsilon}$, $\vec{\varepsilon'}$ are incoming and outgoing polarization, $\vec{k}$, $\vec{k'}$ are incoming and outgoing photon momenta, respectively. $\hat{\psi}_c$ creates a hole in the core and $\hat{\psi}_v^\dagger$ creates an electron in the valence state.

These formulas can be straightforwardly derived from the Fermi golden rule

$$w = \frac{2\pi}{\hbar}\left|\langle \mathrm{g}|\Delta H|\mathrm{g}\rangle + \sum_n \frac{\langle \mathrm{g}|\Delta H|n\rangle\langle n|\Delta H|\mathrm{g}\rangle}{E_g + \hbar\omega - E_n}\right|^2 \quad (11)$$

in which the initial state of the solid with the incoming photon is $|\mathrm{g}\rangle = |g\rangle \otimes |\hbar\omega\rangle$, and the intermediate state of the solid (without the photon) is $|n\rangle$. Their energies are

E_g, $\hbar\omega$, and E_n, respectively. The perturbing electromagnetic filed is

$$\Delta H = \frac{e^2}{2m}\sum_i A^2(r_i) + \frac{e}{2m}\sum_i \vec{A}(r_i)\vec{p}_i + \vec{p}_i\vec{A}(r_i), \quad (12)$$

with $\vec{A}$ the quantized photon field $\vec{A}(r) = \sqrt{\frac{\hbar}{2V\epsilon_0\omega}}\left(\vec{\varepsilon}e^{i\vec{k}\vec{r}}a_k + \vec{\varepsilon}^* e^{-i\vec{k}\vec{r}}a_k^\dagger\right)$ and $\vec{p}$ the electron momentum operator $\vec{p} = \hat{\psi}^\dagger(-i\hbar\nabla)\hat{\psi}$

Because the extent of the core state is very small compared to the photon momentum, we may use the dipole approximation, in which the position operator $\mathbf{r}$ is approximated by the atom position in the phase factor ($e^{i\mathbf{kr}} \approx e^{i\mathbf{kR}_a}$). We may also expand the field operator in terms of the spherical harmonics, and the radial solutions of the Dirac equation inside the muffin-tin sphere, i.e.,

$$\hat{\psi}_c = \sum_{m_c,s} \phi_c(r)Y_{l_c,m_c}(\hat{\mathbf{r}})|\chi_s\rangle\, c_{l_c,m_c,s} \quad (13)$$

$$\hat{\psi}_v = \sum_{m_v,s} \phi_v(r)Y_{l_v,m_v}(\hat{\mathbf{r}})|\chi_s\rangle\, d_{l_v,m_v,s}, \quad (14)$$

where $c_{l_c,m_c,s}$ ($d_{l_v,m_v,s}$) annihilates electron in the core (valence) with momentum l_c, m_c (l_v, m_v) and spin s.

The resonant part of the scattering amplitude then becomes

$$F^{res}_{\mathbf{q}} = \frac{r_0\hbar^2}{m}\sum_{n,a} e^{i\mathbf{qR}_a} \sum_{m_c,m_v,m_v',s,s'} \vec{\varepsilon'}^* \cdot \langle\phi_c Y_{l_c,m_c'}|i\nabla|\phi_v Y_{l_v,m_v'}\rangle \otimes \langle\phi_v Y_{l_v,m_v}|i\nabla|\phi_c Y_{l_c,m_c}\rangle \cdot \vec{\varepsilon} \times \quad (15)$$

$$\times \frac{\langle g|c^\dagger_{l_c,m_c',s'} d_{l_v,m_v',s'}|n\rangle\langle n|d^\dagger_{l_v,m_v,s} c_{l_c,m_c,s}|g\rangle}{E_g - E_n + \hbar\omega} \quad (16)$$

The first line (Eq. 15) contains the matrix elements, and the second (Eq. 16) the two-particle correlation function, which we will describe more precisely below.

The matrix elements are computed quite straightforwardly within our *ab-initio* approach, using the solutions of the Schroedinger equation ($\phi(r)Y_{lm}(\hat{\mathbf{r}})$) inside the muffin-tin sphere at a, we compute

$$\mathbf{P}^{a,l_c,l_v}_{m_c,m_v} \equiv \langle\phi_c Y_{l_c,m_c}|i\hbar\nabla|\phi_v Y_{l_v m_v}\rangle = \quad (17)$$

$$i\hbar\langle\phi_c|\frac{d}{dr}|\phi_v\rangle\langle Y_{l_c,m_c}|\vec{e}_r|Y_{l_v m_v}\rangle$$

$$+ i\hbar\langle\phi_c|\frac{1}{r}|\phi_v\rangle\langle Y_{l_c,m_c}|(r\nabla)|Y_{l_v m_v}\rangle, \quad (18)$$

and the integrals of the spherical harmonics

$$I^1_{l_1m_1,l_2m_2} = \langle Y_{l_1,m_1}|\vec{e}_r|Y_{l_2,m_2}\rangle \quad (19)$$

$$I^2_{l_1m_1,l_2m_2} = \langle Y_{l_1,m_1}|r\nabla|Y_{l_2,m_2}\rangle, \quad (20)$$

are evaluated analytically, and are given in Ref. [6] (Appendix A.22, A23) Note that these integrals lead to the dipole selection rules $l_v = l_c \pm 1$.

To compute the two-particle correlation function in Eq. 16, we first define the following imaginary time correlation function

$$G^{l_c,l_v}_{m_c'm_v's',m_cm_vs}(\tau) = -\langle T_\tau c^\dagger_{l_c,m_c',s'}(\tau) d_{l_v,m_v',s'}(\tau) d^\dagger_{l_v,m_v,s} c_{l_c,m_c,s}\rangle, \quad (21)$$

and then we continue it analytically to frequency space $G^{l_c,l_v}(\omega)$, and take the unoccupied part of its spectra, i.e.,

$$\widetilde{G}^{l_c,l_v}(\omega) = -\frac{1}{\pi}\int \frac{f(-x)\mathrm{Im}G^{l_c,l_v}(x)}{\omega - x + i\delta}dx. \quad (22)$$

Namely, it is straightforward to show that $\widetilde{G}^{l_c,l_v}(\omega)$ has the spectral representation

$$\tilde{G}^{l_c,l_v}(\omega) = \sum_{g,n} \frac{\langle g|c^\dagger_{l_c} d_{l_v}|n\rangle\langle n|d^\dagger_{l_v} c_{l_c}|g\rangle}{\hbar\omega + E_g - E_n + i\delta}\frac{e^{-\beta E_g}}{Z} \quad (23)$$

which is exactly the quantity appearing in Eq. 16.

Putting all these terms together, we finally obtain

$$F_{\mathbf{q}}^{res} = \frac{r_0}{m} \sum_{a,m_c,m_v,m_c',m_v',s,s'} e^{i\mathbf{q}\mathbf{R}_a}\, \vec{\varepsilon}'^{*} \cdot \mathbf{P}^{a,l_c,l_v}_{m_c',m_v'} \otimes \mathbf{P}^{a,l_c,l_v\,*}_{m_c,m_v} \cdot \vec{\varepsilon}\, \widetilde{G}^{l_c,l_v}_{m_c' m_v' s', m_c m_v s}(\omega) \tag{24}$$

For the X-ray scattering at the K-edge, the electron is excited from $1s$ core state to the $4p$ valence state. We notice that the $4p$ valence state is very extended as compared to the $1s$, therefore we can treat the $4p$ states as weakly interacting ("within the DFT+EDMFT average potential") and we can thus decouple the interaction between the $4p$ and $1s$ states, to obtain

$$G^{l_c,l_v}(\tau) \approx -\langle T_\tau c^\dagger_{l_c,m_c',s'}(\tau) c_{l_c,m_c,s}\rangle \langle T_\tau d_{l_v,m_v',s'}(\tau) d^\dagger_{l_v,m_v,s}\rangle = G^{l_c}_{m_c m_c'}(-\tau) G^{l_v}_{m_v' m_v}(\tau) \tag{25}$$

Notice that the scattering amplitude has very simple diagrammatic representation displayed in Fig. 3. Notice also that the above decoupling is equivalent to negligence of the vertex corrections in the displayed bubble diagram. The core $1s$ state and valence $3d$ states are both concentrated near the nucleus, and since the $3d$ state is partially occupied, the interaction between the $1s$ state and $3d$ state is very significant, and similar to the interaction within the $3d$ orbitals. Our estimation is that Hubbard interaction U within $3d$ shell and between $3d$ and $1s$ are almost equal. The spectral function of the $1s$ orbital is therefore not just a Kronecker-delta function at the core energy ε_c, but it should be computed in the presence of the partially occupied $3d$ states. This is so-called dressed Green's function, therefore we plotted $1s$ propagator in Fig. 3 with the tick black line.

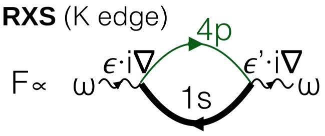

FIG. 3: **Feynman diagram** representation of the resonant X-ray scattering on K-edge, where valence state is treated as non-interacting.

The interaction between $3d$ and $1s$ states can be expanded in terms of Slater integrals, and has both the density-density and Hund's type terms. Here we concentrate on density-density term, which is of the order of $U_{ch} \approx 7\,\text{eV}$, as compared to the Hund's terms, which are of the order of 1 eV. We thus add to the DMFT Hamiltonian (H_{dd}) the Hamitonian of the $1s$ core states, to obtain

$$H = H_{dd} + \varepsilon_c(n_{1s} - 2) + U_{ch}(n_{1s} - 2)(n_{3d} - \langle n_{3d}\rangle) \tag{26}$$

where ε_c is the average core energy, and the constants and potential terms were chosen such that in the absence of $1s$ core-hole $H = H_{dd}$. For convenience, we will perform the particle hole transformation so that $c^\dagger_{1s}$ creates a hole in $1s$ state and c_{1s} annihilates the hole. Then the transformed Hamiltonian becomes

$$H = H_{dd} - c^\dagger_{1s}(\varepsilon_c + U_{ch}(n_{3d} - \langle n_{3d}\rangle))c_{1s} \tag{27}$$

Note that due to the hole notation, the energies appear with an extra minus sign.

As time evolves, the local occupancy in the solid is changing, and it fluctuates between various atomic configurations. The average occupancy $\langle n_{3d}\rangle$ of the Ni atom in $RNiO_3$ is around $\langle n_{3d}\rangle \approx 8$, but there is finite probability for other occupancies, such as $3d^7$ and $3d^9$. We sample such time evolution numerically with the continuous time quantum Monte Carlo method [8].

When the core-hole is created in the $1s$ orbital, the Hamiltonian suddenly changes from $H_{dd} \to H_{dd} - c^\dagger_{1s}c_{1s}(\varepsilon_c + U_{ch}(n_{3d} - \langle n_{3d}\rangle))$, according to Eq. 27. We thus need to simulate a type of non-equilibrium problem.

The core-electron Green's function is given by $G_{1s}(\tau - \tau') = -\langle T_\tau c_{1s}(\tau') c^\dagger_{1s}(\tau)\rangle$ and can be expressed with the functional field integral as

$$G_{1s}(\tau_s - \tau_e) = \frac{1}{Z}\int \mathcal{D}[\psi^\dagger_d \psi_d c^\dagger_{1s} c_{1s}] \exp\left(-S_{dd} - \int_{\tau_s}^{\tau_e}\left(-(\frac{\partial}{\partial\tau}c^\dagger_{1s})c_{1s} - c^\dagger_{1s}(\varepsilon_c + U_{ch}(n_{3d} - \langle n_{3d}\rangle))c_{1s}\right)d\tau\right)\left(-c_{1s}(\tau_e)c^\dagger_{1s}(\tau_s)\right) \tag{28}$$

where c_{1s} and ψ_d are Grassmanns corresponding to the core and $3d$ degrees of freedom. $S_{dd}[\psi^\dagger_d, \psi_d]$ is the action

corresponding to the correlated $3d$ part. As discussed before $c_{1s}^\dagger |g\rangle$ creates a hole in the core, while $c_{1s}|g\rangle = 0$.

The crucial simplification in the CTQMC algorithm comes from the fact that n_{3d} is a good quantum number within each segment of time between two kinks (between the creation or annihilation of $3d$ electron due to hopping into/from the bath), and that the core degrees of freedom c have no hybridization terms, which would require insertion of new kinks. We can use standard manipulations for the functional integrals to rewrite

$$G_{1s}(\tau_s - \tau_e) = \frac{1}{Z}\int \mathcal{D}[\psi_d^\dagger \psi_d] e^{-S_{dd}} \frac{\int \mathcal{D}[c_{1s}^\dagger c_{1s}] \exp\left(-\int c_{1s}^\dagger(\tau_1) A(\tau_1,\tau_2) c_{1s}(\tau_2) d\tau_1 d\tau_2\right) c_{1s}^\dagger(\tau_s) c_{1s}(\tau_e)}{\int \mathcal{D}[c_{1s}^\dagger c_{1s}] \exp\left(-\int c_{1s}^\dagger(\tau_1) A(\tau_1,\tau_2) c_{1s}(\tau_2) d\tau_1 d\tau_2\right)} \quad (29)$$

$$= \frac{1}{Z}\int \mathcal{D}[\psi_d^\dagger \psi_d] e^{-S_{dd}} \left(-\frac{\delta}{\delta A(\tau_s,\tau_e)}\right) \ln \int \mathcal{D}[c_{1s}^\dagger c_{1s}] \exp\left(-\int c_{1s}^\dagger(\tau_1) A(\tau_1,\tau_2) c_{1s}(\tau_2) d\tau\right) \quad (30)$$

$$= \frac{1}{Z}\int \mathcal{D}[\psi_d^\dagger \psi_d] e^{-S_{dd}} \left(-\frac{\delta}{\delta A(\tau_s,\tau_e)}\right) \ln \frac{1}{Det[A]} = \frac{1}{Z}\int \mathcal{D}[\psi_d^\dagger \psi_d] e^{-S_{dd}} \frac{\delta}{\delta A(\tau_s,\tau_e)} \mathrm{Tr}\ln(A) \quad (31)$$

$$= \frac{1}{Z}\int \mathcal{D}[\psi_d^\dagger \psi_d] e^{-S_{dd}} \left(A^{-1}\right)_{\tau_e,\tau_s} \quad (32)$$

$$= \frac{1}{Z}\int \mathcal{D}[\psi_d^\dagger \psi_d] e^{-S_{dd}} \left(\delta(\tau_1-\tau_2)(-\frac{\partial}{\partial \tau_1} - \varepsilon_c - U_{ch}(n_{3d} - \langle n_{3d}\rangle))\right)^{-1}_{\tau_e,\tau_s} \quad (33)$$

where

$$A(\tau_1,\tau_2) = \delta(\tau_1-\tau_2)(-\frac{\partial}{\partial \tau_1} - \varepsilon_c - U_{ch}(n_{3d}(\tau_2) - \langle n_{3d}\rangle))$$

and A vanishes, unless $\tau_s \le \tau_1, \tau_2 \le \tau_e$.

In CTQMC algorithm, we are visiting configurations with probability proportional to $e^{-S_{dd}}/Z$, hence to compute the core Green's function G_{1s}, we just need to evaluate the value of the function A^{-1} on each Monte Carlo configuration (for each visited Feynman diagram), and then we average $\langle A^{-1}\rangle$ over the Metropolis chain.

In the atomic limit, n_{3d} is fixed to the atomic valence over the entire time evolution, and thus the core-Green's function in frequency space is simply given by

$$G_{1s}(i\omega_n) = \frac{1}{i\omega_n - \varepsilon_c - U_{ch}(n_{3d} - \langle n_{3d}\rangle)} \quad (34)$$

but since n_{3d} is fixed, its temporary value is equal to the average ($n_{3d} = \langle n_{3d}\rangle$), hence the core-hole interaction becomes irrelevant.

Another limit, which is easy to evaluate, is the short time (large frequency) limit. In this case both τ_s and τ_e occur very likely on the same time interval, before the hybridization kink intervenes. Let's call the atomic state in this time interval $|m\rangle$, and the $3d$ occupancy is then constant over that interval, and given by the occupancy of the atomic state $\hat{n}_{3d}|m\rangle = n_{3d}(m)|m\rangle$. The probability that τ_s or τ_e fall into the interval with state $|m\rangle$ is probability for that state (P_m), hence the short time limit is $G(i\omega) \approx \sum_m P_m/(i\omega - \varepsilon_c - U_{ch}(n_{3d}(m) - \langle n_{3d}\rangle))$, and $n_{3d}(m)$ is the occupancy of the $3d$ orbital in atomic state $|m\rangle$.

When the valence in $3d$ orbitals fluctuates slowly, but it is not frozen, we can expect nontrivial effects, because n_{3d} can be found almost constat but not equal to the average valence over an extended period of time. This happens in correlated insulators, such as $RNiO_3$, which has non-negligible probability for $n_{3d} = 7$ and $n_{3d} = 9$ configurations.

In Fig.3a-b of the main text we showed the spectral function of both the $1s$ and the $4p$ orbitals on Ni_1 and Ni_2 atoms. Their convolution according to Feynman diagram in Fig. 3 gives the scattering amplitude, and its imaginary part at $\mathbf{q} = 0$ gives resonant X-ray absorption. We show in Fig. 4 the high-resolution X-ray absorption from Ref. [14], and the computed absorption using the above derived formulas. We compare it to the simplest approach in which the core-hole interaction is neglected. We see in Fig. 4b that the addition of the core-hole interaction brings two new peaks in the absorption lineshape. One appears roughly U_{ch} below the main peak, and one U_{ch} above the main peak, which is a consequence of multiple peak spectra of the $1s$ core spectral function. These additional peaks can clearly be resolved in the measured high-resolution absorption in Fig. 4a.

In Fig. 5 we show all 9 components of the scattering matrix F (defined in Eq. 8), and we atom resolve them. The X-ray absorption is proportional to the imaginary part of the trace of the above matrix and averaged over both Ni atoms. We notice that the diagonal components are an order of magnitude larger than the off-diagonal parts. The contribution of the Ni_1 and Ni_2 atoms are split by approximately 1.5 eV, in which 0.7 eV comes from the split of the core levels, and another 0.8 eV from the split of the $4p$ states. The imaginary parts are related

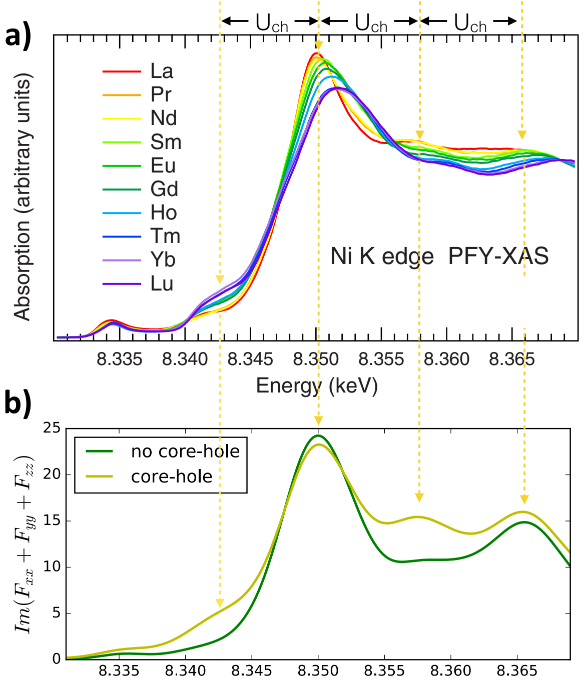

FIG. 4: **X-ray Absorption :** a) Measured high-resolution X-ray absorption from Ref. [14]. b) Calculated X-ray absorption of $NdNiO_3$. The arrows mark the additional peaks due to core-hole interaction, which are separated for approximately $U_{ch} \approx 7eV$.

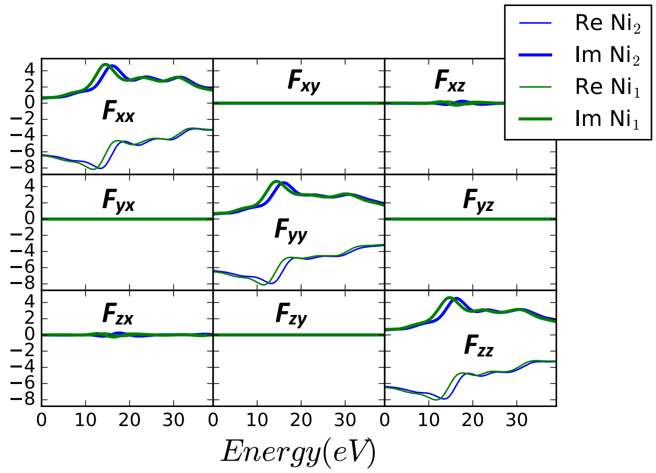

FIG. 5: **Scattering factor:** The energy dependent matrix of the scattering factor.

to the above displayed X-ray absorption curve, and the real parts are Kramars-Kronig related to the imaginary parts.

The xy and yz components vanish for all scattering wave vectors with even $h+k+l$, which is true for all magnetic Bragg peaks. This is because there are two Ni_1 and two Ni_2 atoms per unit cell, related by the glide mirror symmetry ($y \to -y$ mirror and $(1/2, 1/2, 1/2)$ shift). Consequently, the local Green's function on the two equivalent atoms are related by $G^{(1)}_{xy} = -G^{(2)}_{xy}$ and $G^{(1)}_{zy} = -G^{(2)}_{zy}$, and since the scattering factor for these two equivalent atoms is proportional to $G^{(1)}_{ab} + e^{i\pi(h+k+l)} G^{(2)}_{ab}$, these off-diagonal components vanish exactly. The xz component is non-zero, but it is quite small compared to diagonal parts, which is consistent with the fact that the $\sigma - \pi'$ intensity is two orders of magnitude smaller than $\sigma - \sigma'$ scattering intensity [15, 16].

Very recently, the X-ray scattering profile has also been interpreted in terms of the LDA+U magnetic solution in the bond-order structure [13]. In contrast to our results, LDA+U calculation of the scattering factor found substantial off-diagonal components and non-vanishing xy and zy components. This could only come from large symmetry breaking in LDA+U, which is not consistent with the $P2_1/n$ space group. Such large non-sphericity of the $4p$ states is inconsistent with the fact that $\sigma - \pi$ intensity is two orders of magnitude smaller than $\sigma - \sigma$ intensity [15, 16].

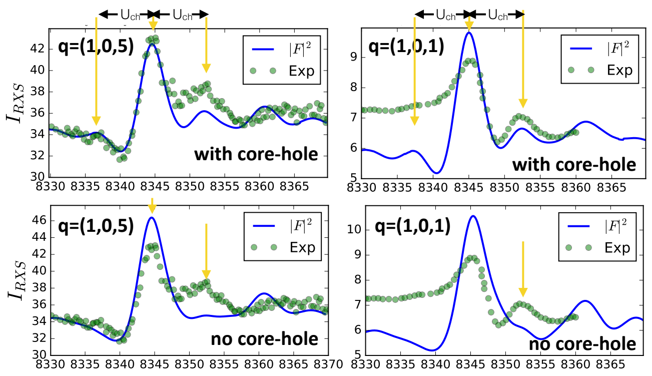

FIG. 6: **X-ray intensity:** Measured and computed X-ray scattering intensity with and without core-hole interaction. Experimental data are reproduced from Ref. 17 and from Ref. 13.

In Fig. 6 we display the $\sigma - \sigma'$ scattering X-ray intensity for Bragg peaks $(1, 0, 5)$ and $(1, 0, 1)$ computed with and without core-hole interaction. We notice that since the off-diagonal components of F are small, and diagonal components are similar, the scattering matrix is approximately spherically symmetric and therefore very weakly depends on azimuthal angle, as also seen in experiment [15].

To understand this result, we note that there are two Ni_1 atoms at position $(0, 0, 0)$ and $(1/2, 1/2, 1/2)$ and two Ni_2 atoms at $(0, 0, 1/2)$ and $(1/2, 1/2, 0)$ in the crystallographic unit cell (not the magnetic unit cell). The corresponding structure is displayed in Fig.1e of the main text. The phase factors $e^{i\mathbf{qR}}$ ($\mathbf{q}$ is the transfer momentum corresponding to the Bragg peak and $\mathbf{R}$ is position of the Ni atoms) then give the following contribution to the structure factor: for the two Ni_1 ions $F_{(0,0,0)} + F_{(1/2,1/2,1/2)} e^{i\pi(h+k+l)}$ and for the Ni_2

ions $e^{i\pi l}(F_{(0,0,1/2)} + F_{(1/2,1/2,0)} e^{i\pi(h+k+l)})$. Hence when $h + k + l$ is even, and l is odd, we have $F_{(0,0,0)} + F_{(1/2,1/2,1/2)} - (F_{(0,0,1/2)} + F_{(1/2,1/2,0)})$. The two Ni_1 ions are equivalent in their local coordinate axis, but due to the rotations of Ni-O octahedra, $F_{(0,0,0)}$ is somewhat different from $F_{(1/2,1/2,1/2)}$. As explained above, the yz and xy components of $F_{(0,0,0)}$ and $F_{(1/2,1/2,1/2)}$ have opposite sign and subtract, and because the xz component is small, the resulting scattering factor is not very far from a spherically symmetric quantity, i.e., $F_{(0,0,0)} \approx F_{(1/2,1/2,1/2)} = F_{Ni_1}$ and $F_{(0,0,1/2)} \approx F_{(1/2,1/2,0)} = F_{Ni_2}$. Within such approximation, the structure factor is proportional to $2(F_{Ni_1} - F_{Ni_2})$, as has been assumed in previous works [17].

Since the scattering factor F on the two Ni atoms is very similar, but shifted in frequency (see Fig. 5), their difference is approximately proportional to the derivative of F, therefore we expect the scattering intensity to also contain several peaks roughly separated by U_{ch}. Indeed we see that the intensity in Fig. 6 has a small shoulder below the main peak, and the second peak above the main peak both separated by U_{ch}. If we neglect the core-hole interaction, the second peak appears approximately 17 eV above the main peak, which comes from the fact that $4p$ density of states (displayed in Fig.3 of the main text) has the second peak also approximately 17 eV above the main peak, and is roughly constant 7 eV above the main peak, resulting in constant intensity at around 8352 eV, which is 7 eV above the main peak. Our calculation thus show that, in contrast to Ref. [13], the side peaks in the measured X-ray intensity can not be explained by the non-interacting or DFT type calculation. Instead, our calculation shows that they require proper treatment of the core-hole interaction.

V. REANALYZING THE NEUTRON POWDER DIFFRACTION EXPERIMENTS AND THEIR CONSISTENCY WITH PROPOSED MAGNETIC MODELS

The high-resolution synchrotron X-ray powder-diffraction experiments conclusively proved that the metal insulator transition in RNiO3 is accompanied by a structural transition from high temperature orthorombic to low temperature monoclinic symmetry [12]. This low temperature monoclinic symmetry is described within the $P2_1/n$ space group, while the orthorhombic symmetry is Pbnm. At the time when the neutron powder diffraction experiments on $NdNiO_3$ were performed [18, 19], there was no conclusive evidence for the low temperature monoclinic structure, and within the experimental resolution of the neutron powder diffraction, there was no support for such a structural transition, therefore the neutron scattering data was analyzed in the orthorhombic Pbnm space group.

The main difference between the two space groups is that within the Pbnm symmetry there is only one type of Ni site, and consequently only one type of NiO_6 octahedra, but within the $P2_1/n$ symmetry there are two nonequivalent Ni ion sites, and consequently two type of NiO_6 octahedra (the expanded and the contracted octahedra). As discussed in the main text of the paper, our theory predicts that Ni ions inside the contracted octahedra carry no magnetic moment. This is different than previously proposed magnetic models for RNiO$_3$, where a finite magnetic moment was considered for all the Ni ions. For early R ions, it was assumed that all Ni ions carry equal magnetic moments, and for some late R-ions, such as $HoNiO_3$ and $YNiO_3$ the best fit gave somewhat different magnitude of the two magnetic moments on the non-equivalent Ni ions (in $YNiO_3$: $m_{Ni_1} = 1.4\mu_B$, $m_{Ni_2} = 0.7\mu_B$ [20] and in $HoNiO_3$: $m_{Ni_1} = 1.4\mu_B$, $m_{Ni_2} = 0.6\mu_B$ [21]). [20, 21]

Here we will concentrate on $NdNiO_3$, for which it was previously argues that all Ni atoms carry the same moments [18, 19], and we will show that the experimental data obtained from neutron powder diffraction can be fitted similarly well with (A) the magnetic models where all the Ni ions have equal magnetic moment and (B) the magnetic models where only half of the ions have a finite magnetic moment (as predicted by our theory). In chapter VII we will show analytically that such near degeneracy of the two types of magnetic models is unavoidable when analyzing the neutron powder diffraction data.

For $NdNiO_3$ two magnetic models were proposed so far [1, 25–27], the first is displayed in Fig. 7(a) and is based on the neutron powder diffraction, while the second is shown in Fig. 10(a) and is based on soft X-ray resonant scattering. As explained above, the neutron powder diffraction data was analyzed in terms of wrong Pbnm symmetry [1], therefore we will reanalyze the neutron powder diffraction data of Ref. 1 by considering the true monoclinic symmetry, found at low temperature below the metal-insulator transition. We will compare the theoretical magnetic model with the other two magnetic models proposed before, as well as several related models, and we will show how consistent they are with the neutron powder diffraction data. In our new refinements we are using (A) the magnetic form factors corresponding to Ni d^8 valence (Ni^{2+}) instead of previously assumed Ni d^7 (Ni^{3+}) and (B) a better approximation for the Nd^{3+} magnetic form factor, as explained in section VI.

The integrated intensity, extracted from a powder neutron diffraction experiment, can be modeled by the formula:

$$I_Q = C \sum_{|\mathbf{Q}| \in Q} LP(\theta)\ [|F^N(\mathbf{Q})|^2 + |\mathbf{F}^M_\perp(\mathbf{Q})|^2] \qquad (35)$$

where C is an arbitrary constant, $LP(\theta)$ is the instrumental parameter called Lorentz factor, $\mathbf{Q} = 2\pi(\frac{h}{a}, \frac{k}{b}, \frac{l}{c})$, and F^N and $\mathbf{F}^M$ are the nuclear (scalar) and magnetic (vector) structure factors, respectively. The first depends on the crystal structure only, while the second is determined from the magnetic model, and its form is explained in detail in section VII. $\mathbf{F}^M_\perp$ represents the component of the

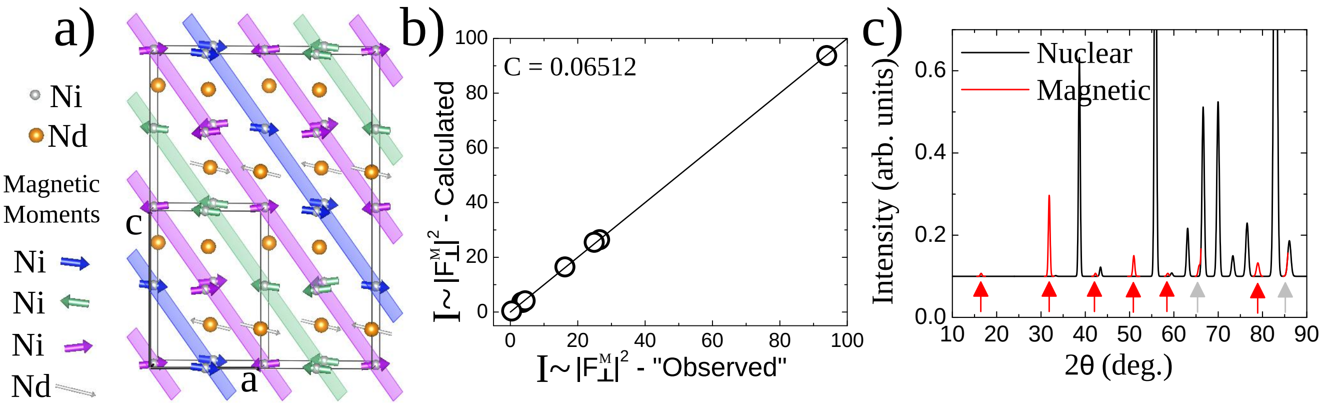

FIG. 7: **Previous results for the Magnetic Model of $NdNiO_3$** (a) Magnetic model proposed in Ref. 1 for the magnetic structure of $NdNiO_3$ at 1.5K, based on powder neutron scattering experiments; (b) Calculated magnetic integrated intensities (I$\sim$ $|F_{\perp}^{M}|^2-$Calculated) versus theoretical magnetic integrated intensities (I$\sim$ $|F_{\perp}^{M}|^2 -$"*Observed*"). The solid line is guide to the eye. The theoretical magnetic integrated intensities were taken from Table II Ref. 1 and they correspond to a magnetic model shown in panel (a); (c) Theoretically simulated neutron powder diffractogram corresponding to a magnetic model shown in panel (a) and a crystal model within the Pbnm symmetry given in Refs. 19. The diffractogram was simulated using FullProf [22–24]. The arrows show the position of the magnetic Bragg reflections. The gray arrows show the Bragg reflections with both nuclear and magnetic contributions.

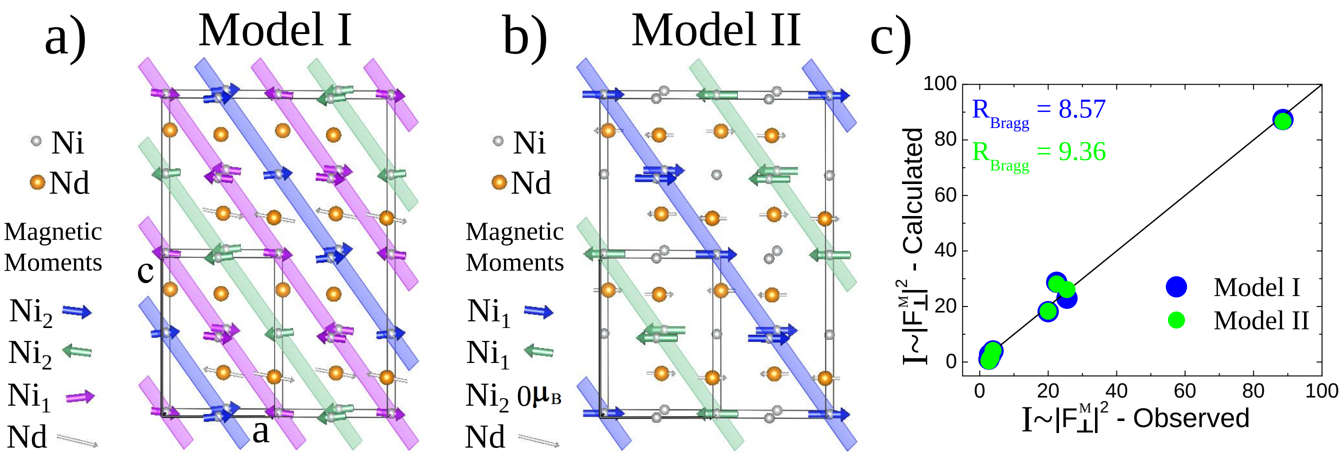

FIG. 8: **Revised Collinear Magnetic Models for $NdNiO_3$** (a) Magnetic model similar with the one proposed in Ref. 1 for the magnetic structure of $NdNiO_3$ at 1.5K, based on new refinements of the powder neutron scattering data; (b) New magnetic model based on theoretical predictions which fits the powder neutron scattering data as well as the proposed model shown in panel (a); (c) Results of the refinement; the solid line is guide to the eye. The experimental magnetic integrated intensities were taken from Table II Ref. 1.

magnetic structure factor perpendicular to the scattering vector **Q**. The sum must be performed over all the (h, k, l) reflections, which have the same scattering angle 2θ within the resolution of the neutron/x-ray instrument ($\sum_{|\mathbf{Q}|\in Q}$). All the data refinements described in this section were performed using FullProf software [22–24]. The earlier version of the same software was also used in Ref. 1 to analyze the original data.

To refine any magnetic model against the experimental magnetic integrated intensities given in Ref. 1, we need to first find the value of the constant C for this particular experiment. As the magnetic integrated intensities for the magnetic model shown in Fig. 7(a) were given in Ref. 1 (in Table II, column I_{calc}) together with the directions and the sizes of all the magnetic moments in this configuration, we can use that information to determine the value of the constant C. The result of this refinement is shown in Fig. 7(b) and the best fit gives $C = 0.06512$. This constant can then be used in our further analysis.

The experimental magnetic integrated intensities are also given in Table II of Ref. 1 as $I_{obs}(\sigma)$. These were extracted from the neutron powder diffractogram assuming

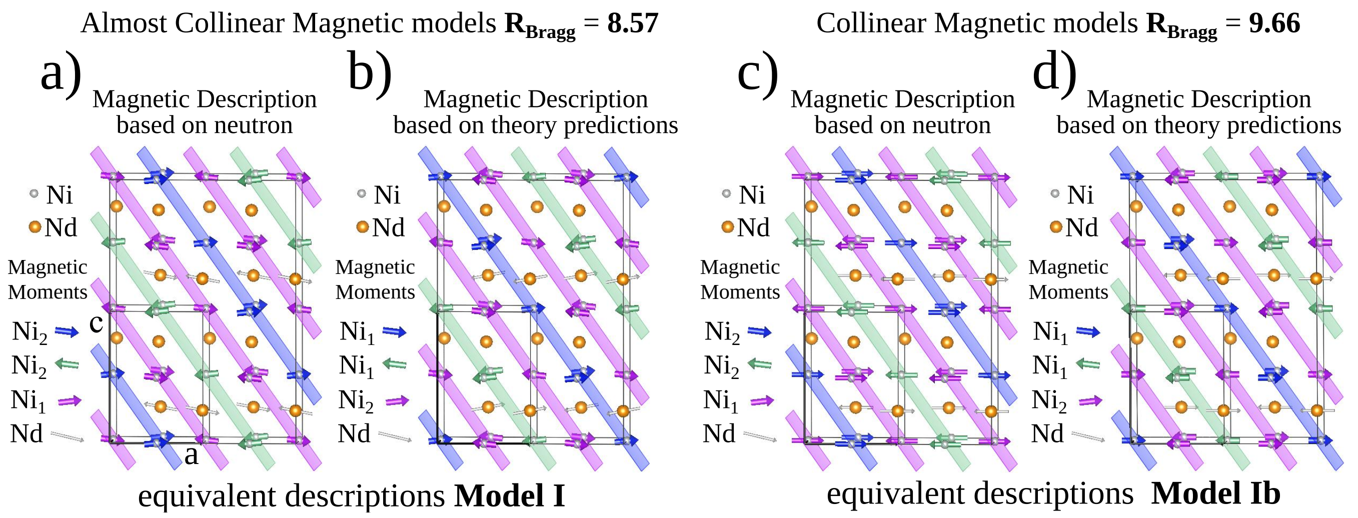

FIG. 9: **Revised Collinear Magnetic Models for $NdNiO_3$** (a) Magnetic model shown in Fig. 8(a); (b) Equivalent magnetic model described based on the theoretical predictions that ferromagnetic layers are situated at the positions of Ni_1 ions and not at the positions of Ni_2 ions as shown in panel (a); (c) Magnetic model based on new refinements of the powder neutron scattering experiments, with constrains that all magnetic moments should be collinear along the a-axis, similar with the first model proposed from neutron scattering Ref. 25; (d) Equivalent magnetic model described based on the theoretical predictions that ferromagnetic layers are situated at the positions of Ni_1 ions and not at the positions of Ni_2 ions as shown in panel (c).

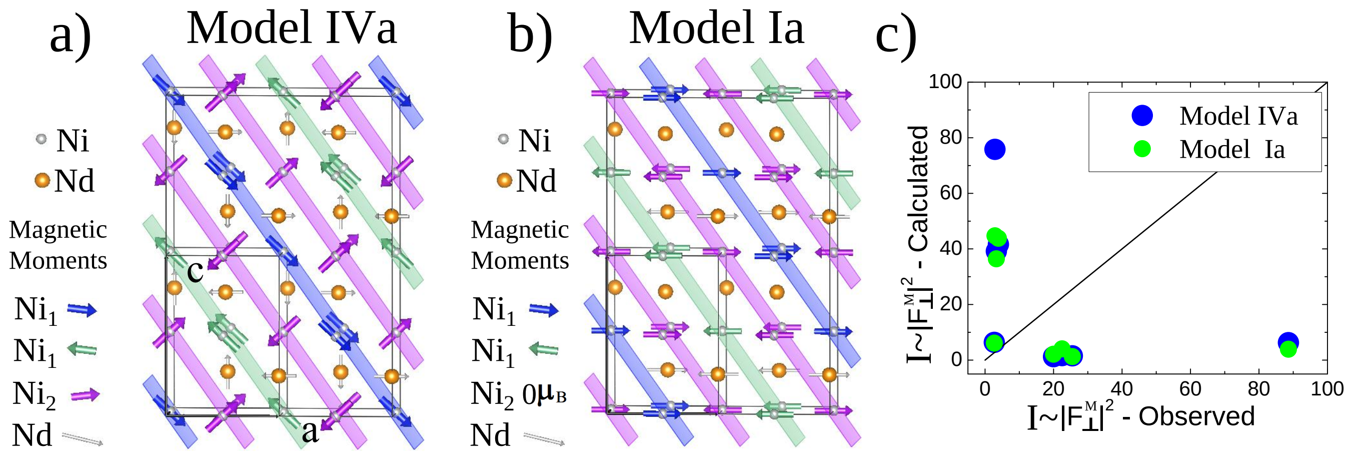

FIG. 10: **Discrepancies of the Noncollinear Magnetic Models proposed for $NdNiO_3$ from soft X-ray resonant scattering experiments** (a) and (b) Magnetic models proposed in Ref. 26, 27 from soft x-ray resonant scattering experiments; (c) Calculated versus observed integrated intensities for the magnetic models shown in panel (a) and (b); as explain in the text, these models give a large discrepancy agreements factors; the solid line is guide to the eye.

a nuclear model based on the Pbnm symmetry. Since the nuclear model used to extract the magnetic integrated intensities was not accurate (Pbnm symmetry instead of $P2_1/n$), we believe that the large disagreement found in Ref. 1 for two magnetic Bragg reflections is due to the slight overlap of these magnetic reflections with the nuclear reflections, as shown in Fig. 7(c) (gray arrows). Due to the overlap of these two magnetic reflections with the nuclear reflections, we can not reliably extract the magnetic integrated intensity from the two magnetic peaks, therefore we did not use these two reflections in our new refinements of the experimental data, but we include them in the final plots of the calculated versus observed integrated intensities, and in calculating the Bragg parameter R_{Bragg} (see below).

The main results of our data analysis are summarized in Figs. 8 and 11. In Fig. 8(a) we show the magnetic model (labeled Model I) which is similar to the model of Ref. 1 based on previous analysis of the same neutron scattering data, with the only difference that our Model

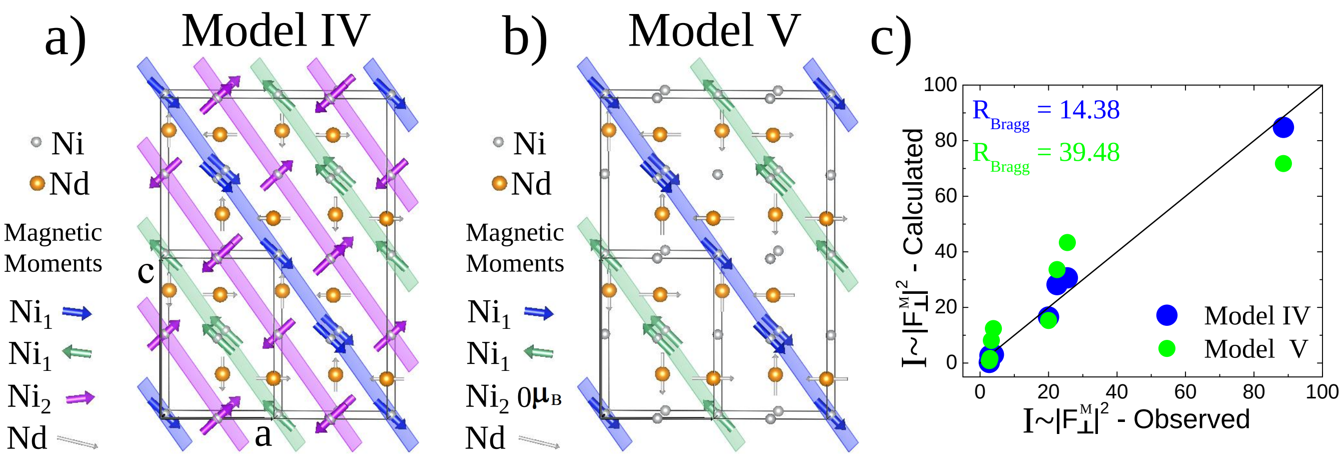

FIG. 11: **Revised Noncollinear Magnetic Models for $NdNiO_3$** (a) Magnetic model based on new refinements of the powder neutron scattering experiments, with constrains on the magnetic moments similar with the ones proposed in Ref. 26, 27 from soft x-ray resonant scattering experiment; (b) New magnetic model based on theoretical predictions; (c) Results of the refinement; the solid line is guide to the eye. The experimental magnetic integrated intensities were taken from Table II Ref. 1.

I has slightly different magnetic moments for Ni^{2+} and Nd^{3+} ions, due to use of the correct monoclinic crystal structure and improved treatment of the atomic magnetic form factors, corresponding to Ni d^8 valence and Nd f^3 valence.

In Fig. 8(b) we show the magnetic configuration (labeled Model II), which we believe is the most likely candidate for the magnetic structure of $NdNiO_3$. It is based on our theoretical result that Ni_2 ions do not carry any magnetic moment, and that Ni_1 ions order ferromagnetically within each $(1, 0, 1)$ plane, and atiferromagnetically between adjacent planes. This model is equivalent to the one displayed in Fig.1i of the main text, except that here all the magnetic moments are rotated clockwise around y-axis for approximately 45 degrees as compared to Fig.1i. In the DMFT theoretical calculation, we did not include spin-orbit coupling on Ni atoms, hence the direction of the magnetic moments in real space can not yet be determined theoretically. Much more precise total energy calculation considering the anisotropy of both Ni and Nd ions would be required to determine the direction. Here we rather resort to the existing neutron powder diffraction data, to determine the direction of the magnetic moments within this model. The results of the refinements are shown in Fig. 8(c). The new theoretical magnetic model (Model II) fits the experimental data as good as the previously proposed mode (Model I). Besides the visual comparison of the calculated and observed integrated intensities, one can use the standard Bragg parameter, defined by

$$R_{Bragg} = 100 \times \frac{\sum(|I_{obs} - I_{calc}|)}{\sum(I_{obs})} \quad (36)$$

to compare the fits. I_{obs} and I_{calc} are the observed and calculated integrated intensities and the sum is over all the reflections measured in the experiment. R_{Bragg} for Model I and Model II are very similar (8.57 vs. 9.36) suggesting that the neutron powder diffraction experiments can not distinguish between these two models. Moreover, the fitted value of the magnetic moment on Ni_1 site is around $1.35\mu_B$, which is similar to theoretically obtained value of $1.2\mu_B$ (see main text), in particularly considering that the theoretical value was obtained at 100 K, which is only half of the critical temperature. A model where half of the Ni ions have zero magnetic moment was also discussed in Ref. 1, but in that work it was found that such model does not fit the neutron data well enough, as its $R_{Bragg} = 24.3$ was found to be much larger than $R_{Bragg} = 9.6$ for the Model I (see Table II in Ref. 1). This discrepancy is likely due to inaccuracies related to the use of the wrong crystal structure and less precise atomic magnetic form factors. For a better understanding of the equivalency between Model I and Model II, in Fig. 9(a) and (b) we show the neutron magnetic model in two different scenarios. In Fig. 9(c) and (d), we show that a purely collinear magnetic model also gives a good agreement with the neutron data Ref. [25].

Based on the soft x-ray resonant scattering experiments [26, 27] Ref. 26 proposed a related magnetic model with non-collinear magnetic configuration, displayed in Fig. 10(a) and named Model IVa. In Ref. 27 the noncolinear Model IVa was compared to a collinear model, which we call Model Ia in Fig. 10(b). The finding of Refs. 26, 27 was that the collinear Model Ia can not describe X-ray scattering results, while the Model IVa fitted their data quite well. Our calculation presented in Fig. 10(c) shows that none of these two models are consistent with the neutron powder diffraction. However, there is a related model, which we call Model IV displayed in Fig. 11(a), which differs from Model IVa by the coupling of the Nd and Ni magnetic moments. In model IV (IVa) they are antiferromagnetically (fer-

romagnetically) aligned. The two configurations (Model IV and Model IVa) are indistinguishable by X-ray scattering experiments. We show in Fig. 11(c) that when the Nd and Ni magnetic moments are antiferromagnetically aligned, but otherwise in similar configuration as discussed in Refs. 26, 27, the fit of the neutron data improves a lot. The Bragg parameter is not as good as for Model I and Model II above, but we still can not exclude it based solely on the neutron scattering refinement. We also tested a related model, which we named Model V (see Fig. 11(b)), where only half on the Ni ions have a finite magnetic moment, while the rest have moments like in Model IV. The refinement gives worse Bragg parameter than for Model IV.

In conclusion, we have shown that: (A) Model II in Fig. 8(b), which is based on DMFT theoretical work, fits the neutron scattering data as well as the related model proposed in Ref. 1. (B) The non-collinear model, which was obtained based on the soft X-ray scattering data [26, 27] and is displayed in Fig. 10(a) does not fit the neutron data, but a closely related models, displayed in 11(a) and (b), fit the neutron data reasonably well.

VI. TECHNICAL DETAILS ON THE NEUTRON POWDER REFINEMENTS

Below we give details of the atomic parameters and magnetic moments in the crystallographic unit cell (not the magnetic unit cell). To obtain all the magnetic moments inside the magnetic unit cell, one needs to use the phase factor corresponding to the propagation vector $\mathbf{k} = (1/2, 0, 1/2)$, i.e., for a related crystallographic unit cell with the origin at the lattice vector $\mathbf{R}$, the magnetic moments are inverted according to the phase-factor $\exp(2\pi\, i\, \mathbf{k}\cdot\mathbf{R})$ [1, 25]. The refinements was performed with the FullProf software [22–24]. The plots were made using VESTA [28].

A. Reproducing published results of Ref. 1 where the crystal structure was believed to be described by Pbnm symmetry

(1) The structural parameters at 1.5K used for the Pbnm symmetry [19]:

a = 5.3834Å, b = 5.3861Å, c = 7.6066Å
$R_{Nia} = (\frac{1}{2}, 0, 0)$, $R_{Nib} = (\frac{1}{2}, 0, \frac{1}{2})$
$R_{Nic} = (0, \frac{1}{2}, \frac{1}{2})$, $R_{Nid} = (0, \frac{1}{2}, 0)$
$R_{Nd1} = (0.9940, 0.0384, 0.25)$
$R_{Nd2} = (0.0060, 0.9616, 0.75)$
$R_{Nd3} = (0.4940, 0.4616, 0.75)$
$R_{Nd4} = (0.5060, 0.5384, 0.25)$

(2) Magnetic moments for the magnetic model [1] presented in Fig. 7(a):

$M_{Nia}(x, y, z) = (+0.88491, 0.00000, -0.10367)$
$M_{Nib}(x, y, z) = (+0.88491, 0.00000, +0.10367)$
$M_{Nic}(x, y, z) = (+0.88491, 0.00000, -0.10367)$
$M_{Nid}(x, y, z) = (+0.88491, 0.00000, +0.10367)$
$M_{Nd1}(x, y, z) = (+2.05341, 0.00000, -0.48956)$
$M_{Nd2}(x, y, z) = (\;\;0.00000, 0.00000, \;\;0.00000)$
$M_{Nd3}(x, y, z) = (\;\;0.00000, 0.00000, \;\;0.00000)$
$M_{Nd4}(x, y, z) = (-2.05341, 0.00000, +0.48956)$
C = 0.06512 (assuming the Pbnm symmetry for the crystal structure [19])

In order to reproduce the results of the refinements given in Ref. 1, we used the magnetic form factors for Ni^{3+} and Nd^{3+} stored internally in FullProf [22–24]. In Fullprof's notation, we used for Ni^{3+} the magnetic form factor MNI3 [$< j_0 >$ approximation] and for Nd^{3+} the magnetic form factor MND3 [$< j_0 >$ approximation].

(3) The peak shape function we used to generate the theoretical powder diffraction profile in Fig. 7(c) was profile number 5 in FullProf, with half-with parameters U=1.161020, V=-0.658240, W= 0.291757 obtained from the FullProf examples for other experiments performed on the same D1B instrument.

B. New results obtained when using the low temperature crystal structure described by P2_1/n symmetry

For all the refinements presented in this work, we used magnetic form factors for Ni^{2+} and Nd^{3+} stored internally in FullProf. In Fullprof's notation, we used for Ni^{3+} the magnetic form factor MNI2 [$< j_0 >$ approximation] and for Nd^{3+} the magnetic form factor JND3 [$< j_0 > +c_2 < j_2 >$ approximation].

The values of the constant C were also refined for each magnetic model, since the value used in Ref. 1 was not accurately determined, due to the fact that the crystal structure used in the refinements was assumed to have the average Pbnm symmetry.

(4) The structural parameters at 50K used for the P2_1/n symmetry [12]:

a = 5.37783Å, b = 5.38846Å, c = 7.60511Å, $\beta = 90.061$
$R_{Nia} = (\frac{1}{2}, 0, 0)$, $R_{Nib} = (\frac{1}{2}, 0, \frac{1}{2})$
$R_{Nic} = (0, \frac{1}{2}, \frac{1}{2})$, $R_{Nid} = (0, \frac{1}{2}, 0)$
$R_{Nd1} = (0.99322, 0.03910, 0.2495)$
$R_{Nd2} = (0.00678, 0.96090, 0.7505)$
$R_{Nd3} = (0.49322, 0.46090, 0.7495)$

$R_{Nd4} = (0.50678, 0.53910, 0.2505)$

Ni_a / Ni_c are inside of what we call contracted octahedra and Ni_b / Ni_d are inside of what we call expanded octahedra. As mentioned in the main text, our theory predicts zero magnetic moment for the Ni_a and Ni_c ions.

(5) Magnetic moments for the magnetic model presented in Fig. 8(a):

$M_{Nia}(x, y, z) = (+0.93102, 0.00000, +0.10050)$
$M_{Nib}(x, y, z) = (+0.93102, 0.00000, -0.10050)$
$M_{Nic}(x, y, z) = (+0.93102, 0.00000, +0.10050)$
$M_{Nid}(x, y, z) = (+0.93102, 0.00000, -0.10050)$
$M_{Nd1}(x, y, z) = (+1.73539, 0.00000, -0.31860)$
$M_{Nd2}(x, y, z) = (\ \ 0.00000, 0.00000, \ \ 0.00000)$
$M_{Nd3}(x, y, z) = (\ \ 0.00000, 0.00000, \ \ 0.00000)$
$M_{Nd4}(x, y, z) = (-1.73539, 0.00000, +0.31860)$
C = 0.06479

(6) Magnetic moments for the magnetic model presented in Fig. 8(b):

$M_{Nia}(x, y, z) = (\ \ 0.00000, 0.00000, 0.00000)$
$M_{Nib}(x, y, z) = (-1.34622, 0.00000, 0.00000)$
$M_{Nic}(x, y, z) = (\ \ 0.00000, 0.00000, 0.00000)$
$M_{Nid}(x, y, z) = (+1.34622, 0.00000, 0.00000)$
$M_{Nd1}(x, y, z) = (+1.24786, 0.00000, 0.00000)$
$M_{Nd2}(x, y, z) = (+1.24786, 0.00000, 0.00000)$
$M_{Nd3}(x, y, z) = (+1.24786, 0.00000, 0.00000)$
$M_{Nd4}(x, y, z) = (+1.24786, 0.00000, 0.00000)$
C = 0.06512

(7) Magnetic moments for the magnetic model presented in Fig. 11(a):

$M_{Nia}(x, y, z) = (+1.06603, 0.00000, +0.99455)$
$M_{Nib}(x, y, z) = (-1.06603, 0.00000, +0.99455)$
$M_{Nic}(x, y, z) = (+1.06603, 0.00000, +0.99455)$
$M_{Nid}(x, y, z) = (+1.06603, 0.00000, -0.99455)$
$M_{Nd1}(x, y, z) = (+0.82296, 0.00000, \ \ 0.00000)$
$M_{Nd2}(x, y, z) = (\ \ 0.00000, 0.00000, -0.82296)$
$M_{Nd3}(x, y, z) = (+0.82296, 0.00000, \ \ 0.00000)$
$M_{Nd4}(x, y, z) = (\ \ 0.00000, 0.00000, -0.82296)$
C = 0.06639

(8) Magnetic moments for the magnetic model presented in Fig. 11(b):

$M_{Nia}(x, y, z) = (\ \ 0.00000, 0.00000, \ \ 0.00000)$
$M_{Nib}(x, y, z) = (-1.16681, 0.00000, +1.16681)$
$M_{Nic}(x, y, z) = (\ \ 0.00000, 0.00000, \ \ 0.00000)$
$M_{Nid}(x, y, z) = (+1.16681, 0.00000, -1.16681)$
$M_{Nd1}(x, y, z) = (+1.33448, 0.00000, \ \ 0.00000)$
$M_{Nd2}(x, y, z) = (\ \ 0.00000, 0.00000, -1.33448)$
$M_{Nd3}(x, y, z) = (+1.33448, 0.00000, \ \ 0.00000)$
$M_{Nd4}(x, y, z) = (\ \ 0.00000, 0.00000, -1.33448)$
C = 0.06618

(9) In the earlier stages of finding the magnetic structure for $NdNiO_3$, a collinear magnetic model was proposed [25] similar with the one shown in Fig. 7(a), but where all the magnetic moments are strictly collinear with the a-axis, see Fig. 9(c). Below we give the refined magnetic moments for the magnetic model labeled Model Ib, shown in Fig. 9(c). The standard Bragg parameter for this model is $R_{Bragg} = 9.66$ which is very similar with R_{Bragg} for the magnetic model shown in Fig. 8(a).

$M_{Nia}(x, y, z) = (+0.93093, 0.00000, 0.00000)$
$M_{Nib}(x, y, z) = (+0.93093, 0.00000, 0.00000)$
$M_{Nic}(x, y, z) = (+0.93093, 0.00000, 0.00000)$
$M_{Nid}(x, y, z) = (+0.93093, 0.00000, 0.00000)$
$M_{Nd1}(x, y, z) = (+1.78159, 0.00000, 0.00000)$
$M_{Nd2}(x, y, z) = (\ \ 0.00000, 0.00000, 0.00000)$
$M_{Nd3}(x, y, z) = (\ \ 0.00000, 0.00000, 0.00000)$
$M_{Nd4}(x, y, z) = (-1.78159, 0.00000, 0.00000)$
C = 0.06479

The only difference between the Model Ib shown in Fig. 9(c) and the Model Ia shown is Fig. 10(b), is that in Model Ia the magnetic moments of Nd and Ni ions are ferromagnetically aligned as opposed to the antiferromagnetic alignment in Model Ib. The reason why we show Model Ia in Fig. 10(b) is to point out that this model is inconsistent with the neutron data, see Fig. 10(c). Model Ia is the magnetic model tested against soft X-ray resonant scattering experiments [26, 27].

(10) In Fig. 10(a) we show the magnetic model labeled Model IVa, which is obtained from Model IV shown in Fig. 11(a) by reversing the direction of the Nd magnetic moments with respect to the direction of the Ni magnetic moments. Model IVa is the magnetic model proposed from soft X-ray resonant scattering experiments [26, 27]. The reason why we show both noncollinear models Figs. 11(a) and 10(a), is to point out that Model IV gives a good agreement with the neutron data as opposed to Model IVa which is inconsistent with the neutron data, see Fig. 10(c).

VII. INSIGHTS INTO THE NEUTRON MAGNETIC SCATTERING

To understand these conclusions to a deeper level, we will here compute analytically the magnetic structure factor for various magnetic models which have been proposed for $RNiO_3$, and discuss the relation between these models. For simplicity, we will use from now on the magnetic unit cell, which is quadrupled in size, namely, $\mathbf{a_{magnetic}} = \mathbf{2} * \mathbf{a}$, $\mathbf{b_{magnetic}} = \mathbf{b}$, $\mathbf{c_{magnetic}} = \mathbf{2} * \mathbf{c}$. We will also choose a unit cell slightly different than above, namely, consistent with the main text, but with the origin shifted compared to the previous chapter. The various models which we will discuss are displayed in Fig. 12.

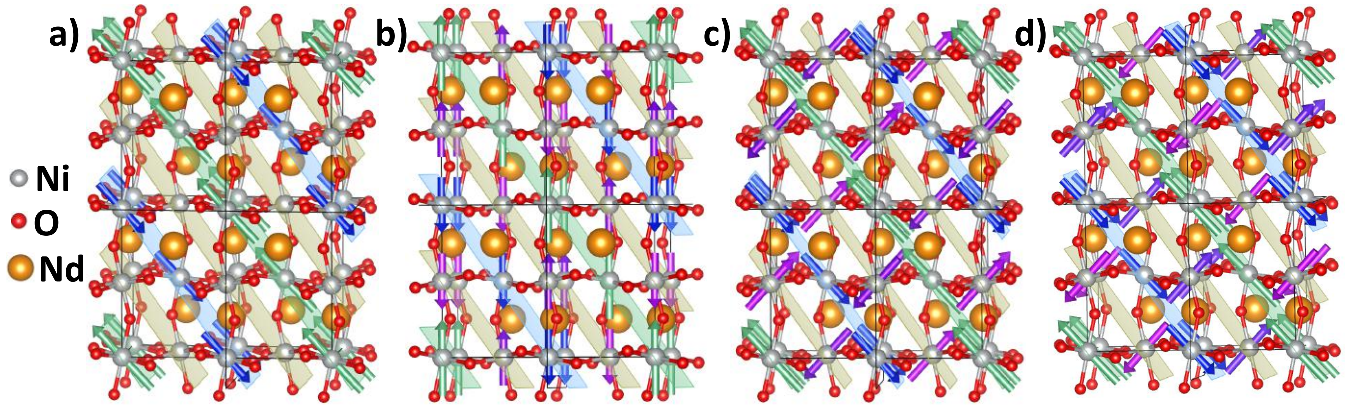

FIG. 12: **Magnetic Configurations:** a) the configuration suggested by the DFT+EDMFT theory, except that here for clarity we orient moments in (-1,0,1) direction, b) the model best fitted to neutron scattering in $NdNiO_3$ (like Fig. figGLP1 above), but for clarity we pointed magnetic moments in z rather then x direction; c) model obtained by neutron scattering on $HoNiO_3$; d) the model suggested by resonant soft X-ray scattering on $NdNiO_3$.

The DMFT calculation suggests the magnetic configuration displayed in Fig. 12a, in which the Ni_2 ions (contracted octahedra laying on yellow planes) have no moments, while Ni_1 ions (expanded octahedra laying on blue and green planes) have ferromagnetic configuration, and planes are antiferromagnetically arranged. Based on neutron powder diffraction, the model in Fig. 12b (Fig. 12c) was proposed for $NdNiO_3$ ($HoNiO_3$) in Ref. 1 (Ref. 21)

Here we want to show that neutron scattering on powder can not distinguish between the theoretical magnetic configuration displayed in Fig. 12a and the configuration fitted to neutron data in Ref 1 or Refs. 20, 21, provided that the magnetic moments on Ni and the rare earths are properly scaled, as we will show below.

The magnetic structure factor is computed by the Fourier transform of the magnetic moments:

$$\mathbf{F}_{\mathbf{Q}} = \sum_{\mathbf{R}_i} e^{i\mathbf{Q}\mathbf{R}_i}\mathbf{m}_i \tag{37}$$

where the magnetic moments on magnetic ion are

$$\mathbf{m}_i = \int_{\mathbf{r}\in\mathbf{R}_i} e^{i\mathbf{Q}(\mathbf{r}-\mathbf{R}_i)}\mathbf{m}(\mathbf{r}-\mathbf{R}_i)d^3r \tag{38}$$

and the intensity for magnetic scattering is proportional to the absolute value squared of the transverse component

$$I \propto |\mathbf{F}^M_\perp(\mathbf{Q})|^2 = |\vec{e}_{\mathbf{Q}} \times \mathbf{F}_{\mathbf{Q}} \times \vec{e}_{\mathbf{Q}}|^2, \tag{39}$$

as already explained in Eq. 35.

We will first compute the part of the magnetic structure factor coming from Ni_1 atoms, which have magnetic moments colored in green and blue in Figs. 12a-c. Half of the atoms have positive component of m along z-direction and the other half have negative. We will denote their position in the unit cell with $\mathbf{R}_\uparrow$ and $\mathbf{R}_\downarrow$, respectively. We have

$$\mathbf{R}_\uparrow = \{(0,0,0),(\frac{3}{4},\frac{1}{2},\frac{1}{4}),(\frac{1}{2},0,\frac{1}{2}),(\frac{1}{4},\frac{1}{2},\frac{3}{4})\} \tag{40}$$

$$\mathbf{R}_\downarrow = \{(\frac{1}{2},0,0),(\frac{1}{4},\frac{1}{2},\frac{1}{4}),(0,0,\frac{1}{2}),(\frac{3}{4},\frac{1}{2},\frac{3}{4})\} \tag{41}$$

We wrote these positions in term of the magnetic unit cell, hence the Bragg peaks also need to be specified in the magnetic Brillouin zone. We will denote them by $\mathbf{Q} = 2\pi(h,k,l)$, with h,k,l integers. The contribution of these atoms to the magnetic structure factor is

$$\mathbf{F}^{(1)}_{\mathbf{Q}} = \mathbf{m}^{(1)}_\uparrow(1-e^{i\pi l})(1-e^{i\pi h})(1-e^{i\frac{\pi}{2}(h+2k+l)}) \tag{42}$$

Nonzero contribution is obtained in the magnetic Bragg peaks, which have odd $h = 2n+1$ and odd $l = 2m+1$. In these peaks, the structure factor simplifies to

$$\mathbf{F}^{(1)}_{\mathbf{Q}} = 4\,\mathbf{m}^{(1)}_\uparrow(1-(-1)^{\frac{h+l}{2}+k}) \tag{43}$$

where $(h+l)/2+k = n+m+1+k$ is integer.

Next we compute the magnetic structure factor for Ni_2 atoms, which carry no magnetic moment in theoretical model of Fig. 12a, but have finite value in the other models. For configuration in Fig. 12b,c we find that the atoms with opposite magnetic moments are

$$\mathbf{R}_\uparrow = \{(\frac{1}{4},\frac{1}{2},0),(\frac{1}{2},0,\frac{1}{4}),(0,0,\frac{3}{4}),(\frac{3}{4},\frac{1}{2},\frac{1}{2})\} \tag{44}$$

$$\mathbf{R}_\downarrow = \{(0,0,\frac{1}{4}),(\frac{3}{4},\frac{1}{2},0),(\frac{1}{4},\frac{1}{2},\frac{1}{2}),(\frac{1}{2},0,\frac{3}{4})\} \tag{45}$$

Their contribution to the magnetic structure factor is

$$\mathbf{F}^{(2)}_{\mathbf{Q}} = -\mathbf{m}^{(2)}_\uparrow e^{i\frac{\pi}{2}l}(1-e^{i\pi l})(1-e^{i\pi h})(1-e^{i\frac{\pi}{2}(h+2k-l)}) \tag{46}$$

and give nonzero contribution for odd h and odd l, namely

$$\mathbf{F}^{(2)}_{\mathbf{Q}} = -4\,i\,\mathbf{m}^{(2)}_\uparrow(-1)^{\frac{l-1}{2}}(1-(-1)^{\frac{h-l}{2}+k}) \tag{47}$$

where $(l-1)/2 = m$ and $(h-l)/2+k = n-m+k$ are integers. Moreover, because l is odd we have $(-1)^{(h-l)/2+k} = -(-1)^{(h+l)/2+k}$, therefore

$$\mathbf{F}^{(2)}_{\mathbf{Q}} = -4\, i\, \mathbf{m}^{(2)}_{\uparrow} (-1)^{\frac{l-1}{2}} (1 + (-1)^{\frac{h+l}{2}+k}) \tag{48}$$

A. Ni moments only

For $YNiO_3$ and $PrNiO_3$ the magnetic moment on rare earths ions vanish, therefore the intensities are simply given by the square of the Ni structure factor. For the configuration in Fig. 12a we get

$$I_a = |\mathbf{F}^{(1)}_{\mathbf{Q},\perp}|^2 = 16 |\mathbf{m}^{(1)}_{\perp}|^2 \left[1 - (-1)^{\frac{h+l}{2}+k}\right]^2 \tag{49}$$

The equivalent expression for the configuration in Fig. 12b-c is computed by $I_b = |\mathbf{F}^{(1)}_{\mathbf{Q},\perp} + \mathbf{F}^{(2)}_{\mathbf{Q},\perp}|^2$, which is greatly simplified by the fact that $\mathbf{F}^{(1)}$ is purely real and $\mathbf{F}^{(2)}$ is purely imaginary, hence we get no interference terms, i.e.,

$$I_b = 16 |\mathbf{m}^{(1)}_{\perp}|^2 \left[1 - (-1)^{\frac{h+l}{2}+k}\right]^2 + 16 |\mathbf{m}^{(2)}_{\perp}|^2 \left[1 + (-1)^{\frac{h+l}{2}+k}\right]^2 \tag{50}$$

These two expressions for I_a and I_b are not yet similar. However, the neutron scattering on powder can not distinguish between scattering vector $\mathbf{Q} = (h, k, l)$ and other related vectors $\mathbf{Q} = (\pm h, \pm k, \pm l)$, hence we need to average over those 8 possible scattering vectors. We will show below that when scattering is averaged over these equivalent reflections, the two expressions I_a and I_b can be brought into an equivalent form.

1. *Ni moments along a high symmetry direction*

First we discuss the case in which the magnetic moment points along the high symmetry directions, either in x or z direction, like in Fig. 12b. Since the Ni_1 and Ni_2 moments are colinear, we have $\mathbf{m}^{(1)} = \mathbf{m}^{(2)}$, when evaluating the configuration in Fig. 12b.

Moreover, the perpendicular magnetic moment is

$$\mathbf{m}_{\perp} = |\vec{e}_{\mathbf{Q}} \times \mathbf{m} \times \vec{e}_{\mathbf{Q}}|^2 = \mathbf{m}^2 - (\mathbf{m} \cdot \vec{e}_{\mathbf{Q}})^2 \tag{51}$$

and for the moment in z direction it takes the form

$$\mathbf{m}_{\perp} = \mathbf{m}^2 \left(1 - \frac{(l/c)^2}{(h/a)^2 + (k/b)^2 + (l/c)^2}\right) \tag{52}$$

hence we see that the magnitude of the moment is invariant under the following three transformations $h \to -h$, or, $k \to -k$, or $l \to -l$.

We then average the configuration I_a (in which Ni_2 have no moments) with respect to $h \to -h$, which gives:

$$I_a = 8 |\mathbf{m}_{\perp}|^2 \left[1 - (-1)^{\frac{h+l}{2}+k}\right]^2 + \left[1 - (-1)^{\frac{-h+l}{2}+k}\right]^2 \tag{53}$$

and because h is odd, we can use $(-1)^{\frac{-h+l}{2}+k} = -(-1)^{\frac{h+l}{2}+k}$, therefore

$$I_a = 8 |\mathbf{m}_{\perp}|^2 \left[1 - (-1)^{\frac{h+l}{2}+k}\right]^2 + \left[1 + (-1)^{\frac{h+l}{2}+k}\right]^2 \tag{54}$$

But this expression I_a is the same as expression for I_b in Eq. 50 if m in configuration I_a is scaled by $\sqrt{2}$. We thus conclude that if Ni magnetic moment points along the high symmetry direction (like in Fig. 12b), the neutron scattering on powder can not distinguish between the configuration in Fig. 12b, and the configuration similar to Fig. 12a, in which Ni_2 atoms have no moments, while Ni_1 moments point in the same high symmetry direction.

2. *Ni moments along* $(1, 0, 1)$ *and* $(-1, 0, 1)$ *direction*

Next we will discuss the configuration in which the moment $\mathbf{m}^{(1)}$ is along $(-1, 0, 1)$ direction, while the moment $\mathbf{m}^{(2)}$ is along $(1, 0, 1)$ direction (like in Figs. 12a,c). This configuration was proposed for $HoNiO_3$ [21]. In this case the expression for the perpendicular moment $|\mathbf{m}^{(i)}_{\perp}|^2 = \mathbf{m}^2 - (\mathbf{m}^{(i)} \cdot \vec{e}_{\mathbf{Q}})^2$ has the following properties

$$|\mathbf{m}^{(1)}_{\perp}(-h, k, l)|^2 = |\mathbf{m}^{(2)}_{\perp}(h, k, l)|^2 \tag{55}$$

$$|\mathbf{m}^{(1)}_{\perp}(h, k, -l)|^2 = |\mathbf{m}^{(2)}_{\perp}(h, k, l)|^2 \tag{56}$$

$$|\mathbf{m}^{(1)}_{\perp}(h, -k, l)|^2 = |\mathbf{m}^{(1)}_{\perp}(h, k, l)|^2 \tag{57}$$

$$|\mathbf{m}^{(2)}_{\perp}(h, -k, l)|^2 = |\mathbf{m}^{(2)}_{\perp}(h, k, l)|^2 \tag{58}$$

Averaging over $\pm k$ is not needed, as both I_a and I_b expressions are invariant under this transformation. Averaging over $h \to -h$ of expression I_a gives

$$\begin{aligned} I_a \;=\; & 8 |\mathbf{m}^{(1)}_{\perp}(h, k, l)|^2 \left[1 - (-1)^{\frac{h+l}{2}+k}\right]^2 \\ +\; & 8 |\mathbf{m}^{(1)}_{\perp}(-h, k, l)|^2 \left[1 - (-1)^{\frac{h+l}{2}+k-h}\right]^2 \end{aligned} \tag{59}$$

hence

$$\begin{aligned} I_a \;=\; & 8 |\mathbf{m}^{(1)}_{\perp}(h, k, l)|^2 \left[1 - (-1)^{\frac{h+l}{2}+k}\right]^2 \\ +\; & 8 |\mathbf{m}^{(2)}_{\perp}(h, k, l)|^2 \left[1 + (-1)^{\frac{h+l}{2}+k}\right]^2 \end{aligned} \tag{60}$$

We again found that this averaged intensities I_a is equivalent to the intensity I_b from Eq. 50 if the moment in I_a

is increased for $\sqrt{2}$. We can therefore conclude that neutron scattering on powder should give exactly the same intensities for model Fig. 12c and Fig. 12a.

The above expressions are sufficient for those rare earth atoms that carry no magnetic moment($YNiO_3$ and $PrNiO_3$), but for others we need to include rare earth magnetic moments.

B. Rare earth moments with different propagating vector

We will start with $HoNiO_3$, in which the neutron scattering was fit to the model in which Ho atoms had propagation vector $(0,0,0)$ in terms of the non-magnetic unit cell. In this case, Ho atoms with one orientation of the magnetic moment have positions

$$\mathbf{R}_{\uparrow} = \{(\frac{x}{2}, y, \frac{z}{2}), (\frac{x}{2}+\frac{1}{2}, y, \frac{z}{2}), (\frac{x}{2}, y, \frac{z}{2}+\frac{1}{2}), (\frac{x}{2}+\frac{1}{2}, y, \frac{z}{2}+\frac{1}{2}), \tag{61}$$

$$(\frac{x}{2}+\frac{1}{4}, -y+\frac{1}{2}, \frac{z}{2}+\frac{1}{4}), (\frac{x}{2}-\frac{1}{4}, -y+\frac{1}{2}, \frac{z}{2}+\frac{1}{4}), (\frac{x}{2}+\frac{1}{4}, -y+\frac{1}{2}, \frac{z}{2}-\frac{1}{4}), (\frac{x}{2}-\frac{1}{4}, -y+\frac{1}{2}, \frac{z}{2}-\frac{1}{4})\} \tag{62}$$

and those with the opposite moments have positions

$$\mathbf{R}_{\downarrow} = \{(-\frac{x}{2}, -y, -\frac{z}{2}), (-\frac{x}{2}-\frac{1}{2}, -y, -\frac{z}{2}), (-\frac{x}{2}, -y, -\frac{z}{2}-\frac{1}{2}), (-\frac{x}{2}-\frac{1}{2}, -y, -\frac{z}{2}-\frac{1}{2}), \tag{63}$$

$$(-\frac{x}{2}-\frac{1}{4}, y-\frac{1}{2}, -\frac{z}{2}-\frac{1}{4}), (-\frac{x}{2}+\frac{1}{4}, y-\frac{1}{2}, -\frac{z}{2}-\frac{1}{4}), (-\frac{x}{2}-\frac{1}{4}, y-\frac{1}{2}, -\frac{z}{2}+\frac{1}{4}), (-\frac{x}{2}+\frac{1}{4}, y-\frac{1}{2}, -\frac{z}{2}+\frac{1}{4})\} \tag{64}$$

The corresponding structure factor of Ho atoms is

$$\mathbf{F}^{Ho}_{\mathbf{Q}} = -2i(1+e^{i\pi h})(1+e^{i\pi l})\mathbf{m}\,[\sin(\pi(hx+2ky+lz)) + \sin(\pi(hx-2ky+lz)+\pi(h/2+k+l/2))] \tag{65}$$

We notice that Ni contributions are nonzero only for odd h and odd l, while Ho contribution vanish for these magnetic Bragg peaks. Since Ho magnetic ions contribute to different Bragg peaks than Ni ions, their intensities can be calculated separately. We can therefore conclude that the above calculation, which does not include rare earth moments, is valid for Ni magnetic Bragg peaks in $HoNiO_3$.

C. Rare earth moments with $\mathbf{q} = (1,0,1)$ propagating vector

Next we discuss the more complex case of rare earth nickelate, such as $NdNiO_3$, where rare earth and Ni moments were assigned the same propagating vector. Such examples are shown in Fig. 13.

We first evaluate the neutron scattering factor for Nd moments in Fig. 13b. The following atoms have moment in the positive direction as compared to Ni atoms

$$\mathbf{R}_{\uparrow} = \{(\frac{x}{2}, y, \frac{z}{2}), (\frac{x}{2}+\frac{1}{2}, y, \frac{z}{2}+1/2), (-\frac{x}{2}+\frac{1}{4}, y+\frac{1}{2}, -\frac{z}{2}-\frac{1}{4}), (-\frac{x}{2}-\frac{1}{4}, y+\frac{1}{2}, -\frac{z}{2}+\frac{1}{4})\} \tag{66}$$

and the following in the negative directions

$$\mathbf{R}_{\downarrow} = \{(\frac{x}{2}+\frac{1}{2}, y, \frac{z}{2}), (\frac{x}{2}, y, \frac{z}{2}+1/2), (-\frac{x}{2}-\frac{1}{4}, y+\frac{1}{2}, -\frac{z}{2}-\frac{1}{4}), (-\frac{x}{2}+\frac{1}{4}, y-\frac{1}{2}, -\frac{z}{2}+\frac{1}{4})\} \tag{67}$$

Here $x = 0.49322$, $y = 0.03910$, $z = 3/4$. The rest of Nd atoms were assigned no moment in Ref. 1, as displayed in Fig. 13b. The resulting neutron form factor evaluates to

$$F^{Nd,(1)}_{\mathbf{Q}} = 4\mathbf{m}^{Nd}e^{2i\pi ky} \times (e^{i\pi(hx+lz)} - e^{-i\pi(hx+lz)-i\pi(\frac{h+l}{2}+k)}) \tag{68}$$

In the theoretical model, displayed in Fig. 13a we assigned the direction of Nd moments to be coupled antiferromagnetically to Ni moments, as our refinements of the neutron data suggested. All Nd magnetic moments which appear in Fig. 13b also appear in Fig. 13a, but in Fig. 13a there are additional magnetic moments on the following atoms

$$\mathbf{R}_{\uparrow} = (-\frac{x}{2}, -y, -\frac{z}{2}), (-\frac{x}{2}-\frac{1}{2}, -y, -\frac{z}{2}-\frac{1}{2}) (\frac{x}{2}-\frac{1}{4}, -y-\frac{1}{2}, \frac{z}{2}+\frac{1}{4}) (\frac{x}{2}+\frac{1}{4}, -y-\frac{1}{2}, \frac{z}{2}-\frac{1}{4}) \tag{69}$$

and

$$\mathbf{R}_{\downarrow} = \{(-\frac{x}{2}-\frac{1}{2}, -y, -\frac{z}{2}), (-\frac{x}{2}, -y, -\frac{z}{2}-1/2), (\frac{x}{2}+\frac{1}{4}, -y-\frac{1}{2}, \frac{z}{2}+\frac{1}{4}), (\frac{x}{2}-\frac{1}{4}, -y+\frac{1}{2}, \frac{z}{2}-\frac{1}{4})\} \tag{70}$$

When the rest of the moments are added, we notice that the second set of atomic positions are related by

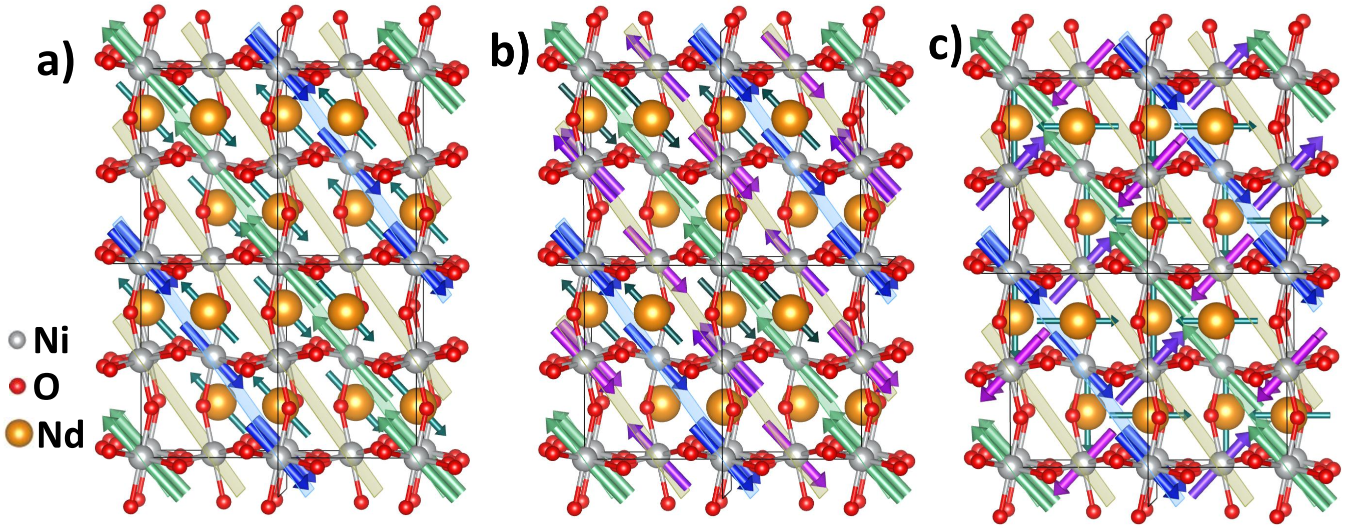

FIG. 13: **Magnetic Configurations:** a) the configuration suggested by the DFT+EDMFT theory, including the rare earth moments, b) the model best fitted to neutron scattering in $NdNiO_3$ (except that moments were oriented along x-direction). c) the model that gives best agreement with the resonant X-ray scattering.

inversion to the first set, and since they have the same moment, their form factor contribution is exactly conjugated to the above form factor, i.e., $F_{\mathbf{Q}}^{Nd,(2)} = (F_{\mathbf{Q}}^{Nd,(1)})^*$.

To compute intensities for magnetic configurations displayed in Figs. 13 we need to add Nd and Ni moments. Previously we computed form factors of Ni atoms. The green and blue moments were summed up to $F_{\mathbf{Q}}^{Ni,(1)}$, while the magenta moments were summed to $F_{\mathbf{Q}}^{Ni,(2)}$. In terms of those, we can write the intensity in Fig. 13b as:

$$I_b = |F_{\mathbf{Q},\perp}^{Ni,(1)} + F_{\mathbf{Q},\perp}^{Ni,(2)} + F_{\mathbf{Q},\perp}^{Nd,(1)}|^2$$

and intensity in Fig. 13a as:

$$I_a = |F_{\mathbf{Q},\perp}^{Ni,(1)} + F_{\mathbf{Q},\perp}^{Nd,(1)} + F_{\mathbf{Q},\perp}^{Nd,(2)}|^2$$

These form factors can be further simplified. We notice that $(h+l)/2+k$ in the relevant magnetic Bragg peaks is an integer number, and can be either even or odd number. We can thus distinguish two cases

(u) $\frac{h+l}{2}+k$ is even: Then

$$F_{\mathbf{Q}}^{Nd,(1)} = 8i\mathbf{m}^{Nd}e^{2i\pi ky}\sin(\pi(hx+lz))$$
$$F_{\mathbf{Q}}^{Nd,(2)} = (F_{\mathbf{Q}}^{Nd,(1)})^* \tag{71}$$

and therefore

$$F_{\mathbf{Q}}^{Nd,(1)} + F^{Nd,(2)} = -16\mathbf{m}^{Nd}\sin(\pi(hx+lz))\sin(2\pi ky)$$

In this case we can also simplify form factor corresponding to Ni moments. The green and blue moments ($F_{\mathbf{Q}}^{Ni,(1)}$) and magenta ($F_{\mathbf{Q}}^{Ni,(2)}$) give

$$F_{\mathbf{Q}}^{Ni,(1)} = 0$$
$$F_{\mathbf{Q}}^{Ni,(2)} = -8i(-1)^{\frac{l-1}{2}}\mathbf{m}^{Ni} \tag{72}$$

The intensity corresponding to Fig. 13b then becomes

$$I_b^u = 64|\mathbf{m}_\perp^{Ni} - (-1)^{\frac{l-1}{2}}\mathbf{m}_\perp^{Nd}e^{2\pi iky}\sin(\pi(hx+lz))|^2 \tag{73}$$

while the intensity corresponding to Fig. 13a is

$$I_a^u = 256(\mathbf{m}_\perp^{Nd})^2\sin^2(2\pi ky)\sin^2(\pi(hx+lz)). \tag{74}$$

(v) $\frac{h+l}{2}+k$ is odd: Then

$$F_{\mathbf{Q}}^{Nd,(1)} = 8\mathbf{m}^{Nd}e^{2i\pi ky}\cos(\pi(hx+lz))$$
$$F_{\mathbf{Q}}^{Nd,(2)} = (F_{\mathbf{Q}}^{Nd,(1)})^* \tag{75}$$

hence

$$F_{\mathbf{Q}}^{Nd,(1)} + F^{Nd,(2)} = 16\mathbf{m}^{Nd}\cos(\pi(hx+lz))\cos(2\pi ky)$$

The Ni moments give rise to the following form factors

$$F_{\mathbf{Q}}^{Ni,(1)} = 8\mathbf{m}^{Ni}$$
$$F_{\mathbf{Q}}^{Ni,(2)} = 0 \tag{76}$$

therefore the corresponding intensities in Figs. 13a,b are

$$I_b^v = 64|\mathbf{m}_\perp^{Ni} + \mathbf{m}_\perp^{Nd}e^{2\pi iky}\cos(\pi(hx+lz))|^2 \tag{77}$$
$$I_a^v = 64|\mathbf{m}_\perp^{Ni} + 2\mathbf{m}_\perp^{Nd}\cos(2\pi ky)\cos(\pi(hx+lz))|^2$$

Here we always need to take the perpendicular component of the magnetic moment, which is given by $\mathbf{m}_\perp = \mathbf{e}_q \times (\mathbf{m} \times \mathbf{e}_q)$. Also note that

$$\mathbf{m}_\perp^1 \cdot \mathbf{m}_\perp^2 = \mathbf{m}^1 \cdot \mathbf{m}^2 - (\mathbf{m}_1 \cdot \mathbf{e}_q)(\mathbf{m}_2 \cdot \mathbf{e}_q) \tag{78}$$
$$(\mathbf{m}_\perp)^2 = \mathbf{m}^2 - (\mathbf{m} \cdot \mathbf{e}_q)^2 \tag{79}$$

If the neutron measurements is done on powder, we need to average over four possibilities, with the transformation $l \to -l$ and $h \to -h$. We notice that when $(h+l)/2+k$ is even $(h-l)/2+k$ is odd, and vice-versa. (similarly for $h \to -h$). We thus see that the scattering on powder measures the average of case u and v, i.e.,

$$I_a = \frac{1}{2}(I_a^u + I_a^v) \tag{80}$$

$$I_b = \frac{1}{2}(I_b^u + I_b^v) \tag{81}$$

D. Moments along high symmetry direction

We will simplify the equations for the case when the magnetic moments point along one of the high symmetry directions, so that $(\mathbf{m}\cdot\mathbf{e}_q)^2$ is equal for h and $-h$ and l and $-l$. Indeed, neutron scattering on $NdNiO_3$ was fit [1] to a model in which all moments were pointing almost entirely in the x (or z) direction.

The result of this averaging is

$$\frac{1}{32}I_b = 2(\mathbf{m}_\perp^{Ni})^2 + (\mathbf{m}_\perp^{Nd})^2 + 2\mathbf{m}_\perp^{Ni}\cdot\mathbf{m}_\perp^{Nd}\cos(2\pi ky)\Big(\cos(\pi(hx+lz)) - (-1)^{\frac{l-2}{2}}\sin(\pi(hx+lz))\Big) \tag{82}$$

$$\frac{1}{32}I_a = (\mathbf{m}_\perp^{Ni})^2 + 2(\mathbf{m}_\perp^{Nd})^2(1+\cos(4\pi ky)\cos(2\pi(hx+lz))) + 4\mathbf{m}_\perp^{Ni}\mathbf{m}_\perp^{Nd}\cos(2\pi ky)\cos(\pi(hx+lz)) \tag{83}$$

This needs to be further averaged over $h \to -h$ and $l \to -l$. The result of such averaging is

$$\frac{1}{32}I_b = 2(\mathbf{m}_\perp^{Ni})^2 + (\mathbf{m}_\perp^{Nd})^2 + 2\mathbf{m}_\perp^{Ni}\cdot\mathbf{m}_\perp^{Nd}\cos(2\pi ky)\cos(\pi hx)\cos(\pi lz) \tag{84}$$

$$\frac{1}{32}I_a = (\mathbf{m}_\perp^{Ni})^2 + 2(\mathbf{m}_\perp^{Nd})^2(1+\cos(4\pi ky)\cos(2\pi hx)\cos(2\pi lz)) + 4\mathbf{m}_\perp^{Ni}\mathbf{m}_\perp^{Nd}\cos(2\pi ky)\cos(\pi hx)\cos(\pi lz) \tag{85}$$

Finally we notice that $\cos(2\pi lz) = \cos(3\pi l/2) = 0$ because $z = 3/4$, and l is odd. We thus obtain

$$\frac{1}{32}I_b = 2(\mathbf{m}_\perp^{Ni})^2 + (\mathbf{m}_\perp^{Nd})^2 + 2\mathbf{m}_\perp^{Ni}\cdot\mathbf{m}_\perp^{Nd}\cos(2\pi ky)\cos(\pi hx)\cos(\pi lz) \tag{86}$$

$$\frac{1}{32}I_a = (\mathbf{m}_\perp^{Ni})^2 + 2(\mathbf{m}_\perp^{Nd})^2 + 4\mathbf{m}_\perp^{Ni}\cdot\mathbf{m}_\perp^{Nd}\cos(2\pi ky)\cos(\pi hx)\cos(\pi lz) \tag{87}$$

If we want to equate the two scattering intensities, we need to require

$$m_{Ni}^a = \sqrt{2}\, m_{Ni}^b \tag{88}$$

$$m_{Nd}^a = \frac{1}{\sqrt{2}} m_{Nd}^b \tag{89}$$

$$\mathbf{m}_{Ni}^a \cdot \mathbf{m}_{Nd}^a = \frac{1}{2}\mathbf{m}_{Ni}^b \cdot \mathbf{m}_{Nd}^b \tag{90}$$

Because x is very close to $\pi/2$ and h is even, $\cos(\pi hx)$ is quite small, hence the contribution from the mixed term between Ni and Nd moments $(\mathbf{m}_{Ni}\cdot\mathbf{m}_{Nd})$ is very small, and hence even when both magnetic moments point in almost the same high-symmetry direction, the scattering intensity in configuration I_a and I_b will be very similar. We thus understand why Model I and Model II in section V (Fig. 8a and Fig. 8b) were almost degenerate. Indeed the ratio of the magnetic moments extracted for Model I and Model II is very close to the above derived $\sqrt{2}$ (see table (5) and table (6) in section VI B). In Model II $m_{Ni}^a \approx 1.35$ and in Model I $m_{Ni}^b \approx 0.93$, which gives the ratio of 1.44, which is very close to $\sqrt{2}$. Similarly $m_{Nd}^a \approx 1.25$ while $m_{Nd}^b \approx 1.74$, which gives the ratio of 1/1.39, again very close to $1/\sqrt{2}$.

VIII. MAGNETIC X-RAY SCATTERING IS CONSISTENT WITH DMFT MODEL

The resonant X-ray scattering on Ni L edge [26] as well as Nd M edge [27] at the Bragg peak $\mathbf{Q} = (1,0,1)$ was performed. The conclusion was that the collinear magnetic configuration displayed in Fig. 12b is not appropriate, and that only non-collinear configuration displayed in Fig. 12d can give azimuthal dependence consistent with X-ray intensity. As shown in chapter V the best fit of the non-colinear model (Model IV) to neutron scattering data is not very good, and the DMFT theory further suggest that only Model V is a viable candidate, which fits neutron data even worse.

Therefore we believe that it is quite likely that the X-ray scattering picked up a signal from multiple domains, as it is known that $RNiO_3$ tend to come in variety of domains [29, 30]. We will show below that if there are two domains, one with the moment pointing in the $(1,0,0)$ direction, and another with the moment in the perpendicular direction, pointing in $(0,0,1)$ in the coordinate system of the first domain, the X-ray scattering on the Model I can explain the X-ray data.

We will approximate scattering on magnetic moments

with a spherical symmetric tensor, and we will take into account only magnetic scattering. None of these approximations are satisfied very well, as Nd carries substantial orbital moment, and it has substantial single ion anisotropy. Nevertheless, we will use this approximation since it was used before to explain the experimental data [27]. For magnetic scattering on such spherical symmetric ion, we have

$$f \propto \mathbf{m}\cdot\vec{\varepsilon}\times\vec{\varepsilon'} \tag{91}$$

where $\vec{\varepsilon}$ ($\vec{\varepsilon'}$) is the incoming (outgoing) polarization. When $\mathbf{Q}$ is along the local x axis, we can write $\mathbf{m}\cdot\vec{\varepsilon}\times\vec{\varepsilon'}$ for all the scattering geometries as

$$f_{\pi,\pi} = -\sin(2\theta)\; m_y \tag{92}$$

$$f_{\pi,\sigma} = \sin\theta\; m_x - \cos\theta\; m_z \tag{93}$$

$$f_{\sigma,\pi} = \sin\theta\; m_x + \cos\theta\; m_z \tag{94}$$

where (m_x, m_y, m_z) is the magnetic moment in the local coordinate system.

Now we want to compute how will the scattering intensity vary with azimuthal angle ψ. Lets suppose that the magnetic moment in the global coordinate axis points in the direction

$$\mathbf{m}_g = (\cos\alpha, 0, \sin\alpha) \tag{95}$$

The scattering in Ref. 27 was performed on $\mathbf{Q} = (1,0,1)$ vector. To orient $\mathbf{Q}$ along x-axis, we rotate coordinate system for $\beta = \arctan(c/a)$ along y axis. Now that the x-axis is along the scattering vector, we can rotate along this new x axis for ψ, to obtain the azimuthal scan. We thus have

$$\mathbf{m} = R_x(\psi)\cdot R_y(\beta)\cdot \mathbf{m}_g = \begin{pmatrix} \cos(\alpha-\beta) \\ -\sin(\alpha-\beta)\sin\psi \\ \sin(\alpha-\beta)\cos\psi \end{pmatrix} \tag{96}$$

Using the expression for the magnetic moment in this local coordinate system, we can evaluate the scattering factor of a single magnetic ion as

$$f_{\pi,\pi} \;=\; \sin(2\theta)\sin(\alpha-\beta)\sin\psi \tag{97}$$

$$f_{\pi,\sigma} \;=\; \sin\theta\;\cos(\alpha-\beta) - \cos\theta\;\sin(\alpha-\beta)\;\cos\psi \tag{98}$$

$$f_{\sigma,\pi} \;=\; \sin\theta\;\cos(\alpha-\beta) + \cos\theta\;\sin(\alpha-\beta)\;\cos\psi \tag{99}$$

This was a contribution of a single atom. Next, we need to compute the scattering factor due to all Nd or Ni atoms in the unit cell. Since we are interested in collinear order, the sum over all equivalent atoms will give just an unimportant pre-factor. For example, for Nd atoms we get

$$F = f^{Nd}\frac{c}{2}[\cos(\pi(hx+2ky+lz)) \tag{100}$$

$$-\cos(\pi(hx-2ky+lz)+\pi(\frac{h+l}{2}+k))] \tag{101}$$

where x, y and z are positions of first Nd atom, and c is an arbitrary constant. For $\mathbf{Q} = (1,0,1)$ vector, it simplifies to

$$F = f^{Nd}\; c\; \cos(\pi(x+z)) \tag{102}$$

To compare with Fig.4 of Ref. 27, we need to compute the ratio

$$\frac{|f^{Nd}_{\pi\pi}|^2 + |f^{Nd}_{\pi\sigma}|^2}{|f^{Nd}_{\sigma\pi}|^2 + |f^{Nd}_{\sigma\sigma}|^2} \equiv R \tag{103}$$

To compare with Fig.3 of Ref. 26, we need to compute

$$|f^{Ni}_{\pi\pi}|^2 + |f^{Ni}_{\pi\sigma}|^2 \equiv I_\pi \tag{104}$$

$$|f^{Ni}_{\sigma\pi}|^2 + |f^{Ni}_{\sigma\sigma}|^2 \equiv I_\sigma \tag{105}$$

If the moments in one domain point in $(1,0,0)$ direction, then $\alpha = 0$ in Eqs. 97-99, and we have

$$f_{\pi,\pi} \;=\; -\sin(2\theta)\sin(\beta)\sin\psi \tag{106}$$

$$f_{\pi,\sigma} \;=\; \sin\theta\;\cos(\beta) + \cos\theta\;\sin(\beta)\;\cos\psi \tag{107}$$

$$f_{\sigma,\pi} \;=\; \sin\theta\;\cos(\beta) - \cos\theta\;\sin(\beta)\;\cos\psi \tag{108}$$

and in the second domain with $\alpha = \pi/2$ the Eqs. 97-99 give

$$f_{\pi,\pi} \;=\; \sin(2\theta)\cos(\beta)\sin\psi \tag{109}$$

$$f_{\pi,\sigma} \;=\; \sin\theta\;\sin(\beta) - \cos\theta\;\cos(\beta)\;\cos\psi \tag{110}$$

$$f_{\sigma,\pi} \;=\; \sin\theta\;\sin(\beta) + \cos\theta\;\cos(\beta)\;\cos\psi \tag{111}$$

The averaging of the intensities over the two domains gives

$$\langle|f_{\pi,\pi}|^2\rangle \;=\; \sin^2(2\theta)\sin^2\psi \tag{112}$$

$$\langle|f_{\pi,\sigma}|^2\rangle \;=\; \sin^2\theta + \cos^2\theta\;\cos^2\psi \tag{113}$$

$$\langle|f_{\sigma,\pi}|^2\rangle \;=\; \sin^2\theta + \cos^2\theta\;\cos^2\psi \tag{114}$$

We can then compute the measured azimuthal intensities. For Nd atoms, the ratio R becomes

$$R = 1 + \frac{\sin^2(2\theta)\sin^2\psi}{\sin^2\theta + \cos^2\theta\;\cos^2\psi} \tag{115}$$

Taking into account that the Bragg condition for Nd M edge requires $\sin\theta \approx 0.7$, we realize that the azimuthal scan from Eq. 115 fits the experimental data of Ref. 27 quite well.

Next we compute the above defined I_π and I_σ intensities on Ni L_3 edge, corresponding to $\sin\theta \approx 0.823$. We get

$$I_\sigma \;=\; \sin^2\theta + \cos^2\theta\;\cos^2\psi \tag{116}$$

$$I_\pi \;=\; \sin^2\theta + \cos^2\theta\;\cos^2\psi + \sin^2(2\theta)\sin^2\psi \tag{117}$$

The azimuthal ψ dependence is identical to the one obtained for non-colinear magnetic configuration in Ref. 26, and agrees well with the measured intensities in Fig. 3 of Ref. 26.

[1] J. L. García-Muñoz, J. Rodríguez-Carvajal, and P. Lacorre. Neutron-diffraction study of the magnetic ordering in the insulating regime of the perovskites $RNiO_3$ (R=Pr and Nd). Phys. Rev. B, 50:978–992, Jul 1994. doi: 10.1103/PhysRevB.50.978. URL http://link.aps.org/doi/10.1103/PhysRevB.50.978.

[2] URL http://hauleweb.rutgers.edu/tutorials/.

[3] G. Kotliar, S. Y. Savrasov, K. Haule, V. S. Oudovenko, O. Parcollet, and C. A. Marianetti. Electronic structure calculations with dynamical mean-field theory. Rev. Mod. Phys., 78:865–951, Aug 2006. doi: 10.1103/RevModPhys.78.865. URL http://link.aps.org/doi/10.1103/RevModPhys.78.865.

[4] Kristjan Haule, Chuck-Hou Yee, and Kyoo Kim. Dynamical mean-field theory within the full-potential methods: Electronic structure of $CeIrIn_5$, $CeCoIn_5$, and $CeRhIn_5$. Phys. Rev. B, 81:195107, May 2010. doi: 10.1103/PhysRevB.81.195107. URL http://link.aps.org/doi/10.1103/PhysRevB.81.195107.

[5] Kristjan Haule and Turan Birol. Free energy from stationary implementation of the DFT + DMFT functional. Phys. Rev. Lett., 115:256402, Dec 2015. doi: 10.1103/PhysRevLett.115.256402. URL http://link.aps.org/doi/10.1103/PhysRevLett.115.256402.

[6] Kristjan Haule and Gheorghe L. Pascut. Forces for structural optimizations in correlated materials within a DFT+embedded DMFT functional approach. Phys. Rev. B, 94:195146, Nov 2016. doi: 10.1103/PhysRevB.94.195146. URL http://link.aps.org/doi/10.1103/PhysRevB.94.195146.

[7] Kristjan Haule. Exact double counting in combining the dynamical mean field theory and the density functional theory. Phys. Rev. Lett., 115:196403, Nov 2015. doi: 10.1103/PhysRevLett.115.196403. URL http://link.aps.org/doi/10.1103/PhysRevLett.115.196403.

[8] Kristjan Haule. Quantum monte carlo impurity solver for cluster dynamical mean-field theory and electronic structure calculations with adjustable cluster base. Phys. Rev. B, 75:155113, Apr 2007. doi: 10.1103/PhysRevB.75.155113. URL http://link.aps.org/doi/10.1103/PhysRevB.75.155113.

[9] P. Blaha, K. Schwarz, G. Madsen, Kvasnicka D., and Luitz J. URL http://www.wien2k.at.

[10] Hyowon Park, Andrew J. Millis, and Chris A. Marianetti. Computing total energies in complex materials using charge self-consistent DFT+DMFT. Phys. Rev. B, 90:235103, Dec 2014. doi: 10.1103/PhysRevB.90.235103. URL http://link.aps.org/doi/10.1103/PhysRevB.90.235103.

[11] Hyowon Park, Andrew J. Millis, and Chris A. Marianetti. Site-selective mott transition in rare-earth-element nickelates. Phys. Rev. Lett., 109:156402, Oct 2012. doi: 10.1103/PhysRevLett.109.156402. URL http://link.aps.org/doi/10.1103/PhysRevLett.109.156402.

[12] J. L. García-Muñoz, M. A. G. Aranda, J. A. Alonso, and M. J. Martínez-Lope. Structure and charge order in the antiferromagnetic band-insulating phase of $NdNiO_3$. Phys. Rev. B, 79:134432, Apr 2009. doi: 10.1103/PhysRevB.79.134432. URL http://link.aps.org/doi/10.1103/PhysRevB.79.134432.

[13] Y. Lu, A. Frano, M. Bluschke, M. Hepting, S. Macke, J. Strempfer, P. Wochner, G. Cristiani, G. Logvenov, H.-U. Habermeier, M. W. Haverkort, B. Keimer, and E. Benckiser. Quantitative determination of bond order and lattice distortions in nickel oxide heterostructures by resonant x-ray scattering. Phys. Rev. B, 93:165121, Apr 2016. doi: 10.1103/PhysRevB.93.165121. URL http://link.aps.org/doi/10.1103/PhysRevB.93.165121.

[14] M. Medarde, C. Dallera, M. Grioni, B. Delley, F. Vernay, J. Mesot, M. Sikora, J. A. Alonso, and M. J. Martínez-Lope. Charge disproportionation in $r\text{nio}_3$ perovskites (r = rare earth) from high-resolution x-ray absorption spectroscopy. Phys. Rev. B, 80:245105, Dec 2009. doi: 10.1103/PhysRevB.80.245105. URL http://link.aps.org/doi/10.1103/PhysRevB.80.245105.

[15] V. Scagnoli, U. Staub, M. Janousch, A. M. Mulders, M. Shi, G. I. Meijer, S. Rosenkranz, S. B. Wilkins, L. Paolasini, J. Karpinski, S. M. Kazakov, and S. W. Lovesey. Charge disproportionation and search for orbital ordering in $NdNiO_3$ by use of resonant x-ray diffraction. Phys. Rev. B, 72:155111, Oct 2005. doi: 10.1103/PhysRevB.72.155111. URL http://link.aps.org/doi/10.1103/PhysRevB.72.155111.

[16] J. E. Lorenzo, J. L. Hodeau, L. Paolasini, S. Lefloch, J. A. Alonso, and G. Demazeau. Resonant x-ray scattering experiments on electronic orderings in $NdNiO_3$ single crystals. Phys. Rev. B, 71:045128, Jan 2005. doi: 10.1103/PhysRevB.71.045128. URL http://link.aps.org/doi/10.1103/PhysRevB.71.045128.

[17] U. Staub, G. I. Meijer, F. Fauth, R. Allenspach, J. G. Bednorz, J. Karpinski, S. M. Kazakov, L. Paolasini, and F. d'Acapito. Direct observation of charge order in an epitaxial $NdNiO_3$ film. Phys. Rev. Lett., 88:126402, Mar 2002. doi: 10.1103/PhysRevLett.88.126402. URL http://link.aps.org/doi/10.1103/PhysRevLett.88.126402.

[18] P. Lacorre, J.B. Torrance, J. Pannetier, A.I. Nazzal, P.W. Wang, and T.C. Huang. Synthesis, crystal structure, and properties of metallic prnio3: Comparison with metallic ndnio3 and semiconducting smnio3. Journal of Solid State Chemistry, 91(2):225 – 237, 1991. ISSN 0022-4596. doi: http://dx.doi.org/10.1016/0022-4596(91)90077-U. URL http://www.sciencedirect.com/science/article/pii/002245969190077U.

[19] J. L. García-Muñoz, J. Rodríguez-Carvajal, P. Lacorre, and J. B. Torrance. Neutron-diffraction study of $R\text{nio}_3$ (R=la,pr,nd,sm): Electronically induced structural changes across the metal-insulator transition. Phys. Rev. B, 46:4414–4425, Aug 1992. doi: 10.1103/PhysRevB.46.4414. URL http://link.aps.org/doi/10.1103/PhysRevB.46.4414.

[20] J. A. Alonso, J. L. García-Muñoz, M. T. Fernández-Díaz, M. A. G. Aranda, M. J. Martínez-Lope, and M. T. Casais. Charge disproportionation in $RNiO_3$ perovskites: Simultaneous metal-insulator and structural transition in ynio_3. Phys. Rev. Lett., 82:3871–3874, May 1999. doi: 10.1103/PhysRevLett.82.3871. URL http://link.aps.org/doi/10.1103/PhysRevLett.82.3871.

[21] M. T. Fernández-Díaz, J. A. Alonso, M. J. Martínez-Lope, M. T. Casais, and J. L. García-Muñoz. Magnetic structure of the honio_3 perovskite. Phys. Rev.

B, 64:144417, Sep 2001. doi: 10.1103/PhysRevB.64.144417. URL http://link.aps.org/doi/10.1103/PhysRevB.64.144417.

[22] Juan Rodrguez-Carvajal. Recent advances in magnetic structure determination by neutron powder diffraction. Physica B: Condensed Matter, 192(1):55 – 69, 1993. ISSN 0921-4526. doi: http://dx.doi.org/10.1016/0921-4526(93)90108-I. URL http://www.sciencedirect.com/science/article/pii/092145269390108I.

[23] T. Roisnel and J. Rodriguez-Carvajal. Winplotr: a windows tool for powder diffraction patterns analysis materials science forum. Proceedings of the Seventh European Powder Diffraction Conference (EPDIC 7), pages 118–123, 2000, Ed. R. Delhez and E.J. Mittenmeijer.

[24] Juan Rodriguez-Carvajal and Thierry Roisnel. Fullprof.98 and winplotr: New windows 95/nt applications for diffraction commission for powder diffraction. International Union for Crystallography, Newletter N20 (May-August), 1998.

[25] J. L. Garca-Muoz, J. Rodrguez-Carvajal, and P. Lacorre. Sudden appearance of an unusual spin density wave at the metal-insulator transition in the perovskites $RNiO_3$ (R= Pr, Nd). EPL (Europhysics Letters), 20(3):241, 1992. URL http://stacks.iop.org/0295-5075/20/i=3/a=009.

[26] V. Scagnoli, U. Staub, A. M. Mulders, M. Janousch, G. I. Meijer, G. Hammerl, J. M. Tonnerre, and N. Stojic. Role of magnetic and orbital ordering at the metal-insulator transition in $ndnio_3$. Phys. Rev. B, 73:100409, Mar 2006. doi: 10.1103/PhysRevB.73.100409. URL http://link.aps.org/doi/10.1103/PhysRevB.73.100409.

[27] V. Scagnoli, U. Staub, Y. Bodenthin, M. García-Fernández, A. M. Mulders, G. I. Meijer, and G. Hammerl. Induced noncollinear magnetic order of Nd^{3+} in $NdNiO_3$ observed by resonant soft x-ray diffraction. Phys. Rev. B, 77:115138, Mar 2008. doi: 10.1103/PhysRevB.77.115138. URL http://link.aps.org/doi/10.1103/PhysRevB.77.115138.

[28] Koichi Momma and Fujio Izumi. *VESTA*: a three-dimensional visualization system for electronic and structural analysis. Journal of Applied Crystallography, 41(3):653–658, Jun 2008. doi: 10.1107/S0021889808012016. URL https://doi.org/10.1107/S0021889808012016.

[29] P. Laffez, O.I. Lebedev, P. Ruello, R. Desfeux, G. Banerjee, and F. Capon. Evidence of strain induced structural change in hetero-epitaxial ndnio3 thin films with metal-insulator transition. Eur. Phys. J. Appl. Phys., 25:25, Dec 2004. doi: 10.1051/epjap:2003087. URL http://dx.doi.org/10.1051/epjap:2003087.

[30] M. H. Upton, Yongseong Choi, Hyowon Park, Jian Liu, D. Meyers, J. Chakhalian, S. Middey, Jong-Woo Kim, and Philip J. Ryan. Novel electronic behavior driving $ndnio_3$ metal-insulator transition. Phys. Rev. Lett., 115:036401, Jul 2015. doi: 10.1103/PhysRevLett.115.036401. URL http://link.aps.org/doi/10.1103/PhysRevLett.115.036401.
